# The Impact of ACEs on BMI: An Investigation of the Genotype-Environment Effects of BMI

**DOI:** 10.3389/fgene.2022.816660

**Published:** 2022-03-07

**Authors:** Karen A. Schlauch, Robert W. Read, Iva Neveux, Bruce Lipp, Anthony Slonim, Joseph J. Grzymski

**Affiliations:** ^1^ Center for Genomic Medicine, Desert Research Institute, Reno, NV, United States; ^2^ Renown Health, Reno, NV, United States

**Keywords:** GWEIS, gene-environment interactions, BMI, adverse child experiences, schizophrenia

## Abstract

Adverse Childhood Experiences are stressful and traumatic events occurring before the age of eighteen shown to cause mental and physical health problems, including increased risk of obesity. Obesity remains an ongoing national challenge with no predicted solution. We examine a subset of the Healthy Nevada Project, focusing on a multi-ethnic cohort of 15,886 sequenced participants with recalled adverse childhood events, to study how ACEs and their genotype-environment interactions affect BMI. Specifically, the Healthy Nevada Project participants sequenced by the Helix Exome+ platform were cross-referenced to their electronic medical records and social health determinants questionnaire to identify: 1) the effect of ACEs on BMI in the absence of genetics; 2) the effect of genotype-environment interactions on BMI; 3) how these gene-environment interactions differ from standard genetic associations of BMI. The study found very strong significant associations between the number of adverse childhood experiences and adult obesity. Additionally, we identified fifty-five common and rare variants that exhibited gene-interaction effects including three variants in the *CAMK1D* gene and four variants in *LHPP*; both genes are linked to schizophrenia. Surprisingly, none of the variants identified with interactive effects were in canonical obesity-related genes. Here we show the delicate balance between genes and environment, and how the two strongly influence each other.

## Introduction

Childhood trauma and adversity has long been linked with a greater risk of negative adult health outcomes (Felitti et al., 1998; McCrory et al., 2011; Merrick et al., 2019; Jones et al., 2020; Park et al., 2020). Adverse Childhood Experiences or Events (ACEs) are defined as traumatic events and unsafe environments occurring in children before the age of 18 (Felitti et al., 1998). The original ACE questionnaire and scoring protocol contains ten Yes/No questions that examine the incidence of emotional, physical, sexual maltreatment, neglect, substance abuse within the household, mental illness in the household, violence, and incarceration of a household member (Felitti et al., 1998).

ACEs range across several of the Social Determinants of Health (SDOH) categories as defined by the Center for Disease Control (CDC), including safe housing, violence, education, income, access to (nutritious) food; all SDOH factors have been shown to have profound impact on individuals’ physical health and quality of life. Numerous national studies indicate the seriousness and incidence of ACEs with prevalence as high as 63.6% of adults experiencing at least one ACE and 16% experiencing at least four ACEs; the CDC reports that at least five of the top ten leading causes of death are associated with ACEs (Merrick et al., 2019; Jones et al., 2020; Park et al., 2020).

Obesity is another challenge for North America (TFAH, 2020). Approximately 70% of adults in the US are considered overweight (BMI ≥ 25 kg/m^2^) or obese (BMI ≥ 30 kg/m^2^), which causes yearly increases in health care expenses (Ward et al., 2021). Each one-unit increase in BMI can increase the cost of healthcare by $253 per patient each year, and those obese individuals have an overall excess healthcare cost of $1,861 per patient per year (Ward et al., 2021). In Nevada, the adult obesity rate is between 30 and 35% (TFAH, 2020). The general rate of obesity is higher in low-to-middle income countries than in high-income countries, highlighting an association of lower income with higher BMI (Levine, 2011; Kim et al., 2018).

There are a number of known environmental and genetic links to obesity. More specifically, associations between ACEs and adult obesity are substantiated by several studies (Felitti et al., 1998; Danese and Tan, 2014; Shields et al., 2016; Merrick et al., 2019; Jones et al., 2020; Wiss and Brewerton, 2020): individuals who report at least one ACE are at a much higher risk of becoming overweight and suffering from adult obesity (Fuemmeler et al., 2009; Davis et al., 2019; Gardner et al., 2019). The California Women’s Health Study found that obese women were 27% more likely to report sexual or physical childhood abuse (Alvarez et al., 2007). In the original ACE cohort studied by Felitti, participants who experienced more than three ACEs had 1.5-fold risk for severe obesity (BMI ≥ 35 kg/m^2^) in adulthood (Felitti et al., 1998); in a more recent study, adults who recalled four or more ACEs were 1.9 times as likely to be severely obese (Anda et al., 2006). Although all mechanisms relating ACEs and obesity are unknown, the most frequently cited in published studies include social disruption, chronic stress, mental health, and socioeconomic status (Wiss and Brewerton, 2020), whereas others include stress-induced overeating, sleep issues, and changes in the gut microbiome (Vámosi et al., 2010; Miller and Lumeng, 2018; Tomiyama, 2019).

Genetic links with obesity have also been well established, and most genetic associations include a “standard” set of obesity-related genes and common variants (Frayling et al., 2007; Scuteri et al., 2007; Loos et al., 2008; Song et al., 2008; Willer et al., 2009; Speliotes et al., 2010; Graff et al., 2013; Namjou et al., 2013; Locke et al., 2015; Rask-Andersen et al., 2017; Iepsen et al., 2018; Sahibdeen et al., 2018; Yengo et al., 2018; Schlauch et al., 2019, 2020; Akbari et al., 2021; Chalazan et al., 2021; Loos and Yeo, 2021). Yet even with large sample sizes, the genetic effects from some of the most recent studies have only been able to explain a small portion of BMI variance, approximately 6% (Yengo et al., 2018; Loos and Yeo, 2021). Here we study how the effects of both genetics and Social Determinants of Health are linked to BMI. While we focus on the impact of ACEs on BMI, we also consider smoking, alcohol consumption, and education as possible influential environmental exposures.

The Healthy Nevada Project (HNP), is an all-comers population health study that was formed in 2016 (Read et al., 2019, 2021; Cirulli et al., 2020; Grzymski et al., 2020; Schlauch et al., 2020). As of October 2021, the HNP includes 43,000 whole-exome sequenced participants who are cross-referenced with up to 16 years of Electronic Health Records (EHR). Additionally, 17,839 participants have responded to a voluntary, retrospective ACEs survey, providing a unique basis to study clinical, sociodemographic, and genetic drivers of BMI.

In this study, we examine links between ACEs and BMI in the HNP cohort. We first establish the profound association between ACEs and BMI in the absence of genetics. We then explore the genome-wide-environment interactions of BMI with ACEs using approximately five million rare and common variants. This genome-wide environment interaction study (GWEIS) examines the genotype-environment (*GxE*) effect of each variant independently, enabling the detection of variants that cause participants with different genotypes to react differently across ACE exposures in terms of BMI. Many of these variants do not associate with BMI levels in a gene-only GWAS, and therefore would not be used in a standard two-step genotype-environment study examining only significant variants of interest.

Most readily available long-term treatments of obesity are not successful, making prevention an essential key to overcoming the disease (LeBlanc et al., 2011; Montesi et al., 2016). Knowledge of potential risk factors, such as ACEs and their modification, becomes an important aspect of disease prevention.

## Methods

### Data Disclosure Statement

In order to minimize unintentional sharing of information that can be used to re-identify private information, a subset of the phenotype data used in this study is available at https://datadryad.org/. Additionally, included are summary statistics of results that support the study’s findings. Please see the Data Availability Statement below.

### IRB and Informed Consent

This study was conducted under a human subject protocol approved by the University of Nevada Institutional Review Board under project #1106618-15. Participants in the Healthy Nevada Project undergo written and informed consent to having genetic information associated with electronic health information (EHR) in a deidentified manner. Inclusion criteria are individuals older than 18 years who can appear in person at an HNP study location to participate in the education and consent process. A copy of the consent can be found at https://healthynv.org/about/consent/. Patient identifiers are not incorporated into the research EHR: the EHR and genetic data are linked in a separate environment via a unique identifier as approved by the IRB.

### The Renown EHR Database

The Renown Health EHR system was instantiated in 2007 on the EPIC system (EPIC System Corporation, Verona, Wisconsin, United States), and contains lab results, diagnosis codes (ICD9/ICD10), and demographic information of approximately 1.8 million hospital patient visits from 2005 to the present date.

### Social Health Determinants Questionnaire

All consenting HNP participants are invited to complete the HNP social health determinants survey. The survey is voluntary and confidential bound through an NIH Certificate of Confidentiality. The survey consists of 103 sociodemographic-related questions including adverse childhood events (ACEs), other traumatic events, education, household income, alcohol use, cigarette use, drug use, and other behavioral patterns. Survey questions for ACEs closely follow the ten questions described previously (Felitti et al., 1998; Shonkoff, 2016; Merrick et al., 2019; Jones et al., 2020). A standard ACE questionnaire and scoring protocol can be found here: https://www.ncjfcj.org/wp-content/uploads/2006/10/Finding-Your-Ace-Score.pdf. Although ACEs are the focus of this study, we also use three other self-reported sociodemographic factors to use as validation: alcohol use, cigarette smoking, and education level. The HNP survey was created on the Survey Monkey platform [www.SurveyMonkey.com], based on the retrospective recall of each participating individual.

### HNP Cohorts

The HNP is an all-comers population health study located in Nevada, with targeted recruitment occurring throughout the state’s urban and rural areas. The project as of October 2021 includes 43,000 participants with a wide range of sociodemographic characteristics. Each participant has cross-referenced electronic medical records, and 17,389 also participated in the HNP Social Health Determinants Questionnaire. In this research, the main focus lies on 11,880 European HNP participants with both BMI medical records and self-reported survey responses of adverse childhood events. We define participants as European based on their genetic admixture (European admixture >0.85, Other ethnicity admixture <0.1) and refer to this cohort as **HNP**_**EU**_. Further, all quality control measures were performed identically on the smaller cohorts: African American (HNP_AA_, African admixture > 0.3, East and South Asian admixture <0.1), and LatinX (HNP_LX_, North American admixture > 0.1, East and South Asian admixture <0.1) cohorts (*N* = 304, *N* = 1,774) respectively. These studies are presented in the Supplementary Materials.

A subcohort of participants suffering from schizophrenia was extracted from the HNP to test the relationship between schizophrenia and ACES, as many of top GWEIS hits were observed in schizophrenia-related genes. Participants with the diagnosis “Schizophrenia” and/or ICD9 code 295.xx (typically 295.90) or ICD10 code F20.9 were included in the schizophrenia subcohort (*N* = 74). Exclusionary criteria for their controls were Psychological Disorders, ICD9 codes 295-306.99.

### Phenotypic Measures

BMI measures for HNP participants were processed as published previously (Schlauch et al., 2020; Read et al., 2021). Briefly, multiple records were agglomerated while taking outlying measures into account. As these processed measures were not normally distributed, processed BMI values were transformed via a rank-based inverse normal transformation with the RankNorm function in **R** and the offset *k* = 0.375 that corresponds to the Blom transform, following other similar studies (Locke et al., 2015; Rask-Andersen et al., 2017). Raw BMI measures are presented as *kg*/*m* ^*2*^. Transformed BMI measures are denoted by BMI_T_. Both raw and transformed BMI values of HNP_EU_ participants are presented in Supplementary Table S1.

### ACEs as an Environmental Exposure

Adverse childhood events (ACEs) were reported as “Yes” or “No” for each of ten types of events based on the retrospective recall of each participating HNP individual. Unanswered questions were recorded as “NA”. Any participant answering at least one of the ten ACE questions was included in this study; i.e., participants were excluded if all ACE questions were left unanswered. Exactly 526 (0.32%) of the questions were missing/blank, stemming from 384 participants, yielding a very small degree of missingness. The ACE score was computed as the sum of affirmative responses a participant reported (Gilbert et al., 2015; Park et al., 2020; Tsai et al., 2020). A participant with two ACEs, for example, encountered two different types of adverse childhood events at least one time each by the age of 18. The number of ACEs was used as a main and interactive effect in linear regression analyses as a whole number between zero and ten.

### Other Environmental Exposures

Available responses for alcohol consumption in the questionnaire are: “Never”, “Monthly or less”, “2-4 times a month”, “2-3 times a week”, “4 or more times a week”, and used as factors in linear regressions. Cigarette smoking was recorded as a whole number denoting the number of cigarettes smoked per day and used as such in regression analyses. Education level was collected as one of these responses: “No High School Diploma”, “High School Diploma”, “GED”, “Some College”, “Associate Degree”, “Bachelor’s Degree”, “Graduate Degree”. These seven responses were used as a seven-element factor in the linear regression. We use these environmental factors to replicate other published genotype-environment (*GxE*) interactions in Europeans.

### Other Statistical Tests

A simple linear regression between the dependent variable BMI and the independent variable ACEs was performed, with age and sex as covariates. ACEs were represented as a whole number between zero and ten. BMI measures were transformed to BMI_T_ as described above. Fisher exact tests were used to compute and examine odds ratios between disease case and control groups (e.g., obesity) as well as ACE case and control groups.

### Sequencing

Sequencing was performed at Helix (CLIA #05D2117342, CAP# 9382893) using a proprietary exome platform called Helix Exome+. This platform combines a medical-grade exome platform with hundreds of thousands additional genomic regions of interest, resembling a microarray backbone (Helix, 2019; Cirulli et al., 2020; Read et al., 2021). Full base pair level coverage histograms demonstrate that more than 90% of the bases have greater than or equal to 20x coverage for popular reference panels including ACMG-73. This assay has also been validated with high reproducibility using high confidence calls from the Platinum genomes (Eberle et al., 2017) and the National Institute of Standards and Technology (NIST) Genome in a Bottle (GIAB) (Zook et al., 2014). All sequencing reads were aligned to GRCh38 and variant calls were made using Sentieon (Helix, 2019; Kendig et al., 2019) following established sequencing-specific quality control metrics and GATK best practices (Helix, 2019; Cirulli et al., 2020; Read et al., 2021).

### Genotype Annotation

Variants were annotated similar to previous HNP studies (Read et al., 2021), using dbSNP build 153 (ftp.ncbi.nlm.nih.gov/snp) and PhenoScanner V2 (Staley et al., 2016; Kamat et al., 2019). Additionally, the Ensembl Variant Effect Predictor v.101 and ClinVar were used for functional characterization. Any variant that was near a gene (within 200 kb base pairs) rather than in it, was referenced with an asterisk in the tables, following results by Thorleiffson and Speliotes (Thorleifsson et al., 2009; Speliotes et al., 2010). Associations not published in these databases are denoted in this manuscript as “not published to the best of our knowledge”.

### Quality Control Processing of Sequencing Data

Rare and common variant calls were processed for quality control similar to previous studies (Anderson et al., 2010; Panoutsopoulou and Walter, 2018; Cirulli et al., 2020; Read et al., 2021). Relationship interference was performed using KING to define first-degree relatives as described previously (Manichaikul et al., 2010; Read et al., 2021). The relative with highest genotyping rate was retained in each relative group (Anderson et al., 2010). Any variants out of Hardy-Weinberg equilibrium (*p* < 1 × 10^−15^) were excluded (Van Hout et al., 2020). Variants with call rate greater than 90% were deemed high quality and retained; only individuals with call rates greater than 70% were included in the analysis (Read et al., 2021). As an additional quality control metric, variants with less than 10 copies of the minor allele were removed (Read et al., 2021). This left 4,876,698 variants of high quality in the HNP_EU_. Variants were not excluded based on ontology type; i.e., all sequencing ontologies (i.e. missense, nonsense, synonymous, indels, frameshifts, etc.) were included. Standard principal component analysis was applied to pruned variants to correct for any population substructure. Statistical models were adjusted by the first five principal components, similar to Read et al., 2021, which yielded a genomic inflation factor of *λ* ≤ 1.07 for all models. (Read et al., 2021).

### Genome-wide by Environment Interaction Study (GWEIS)

Linear regression under the additive genetic model was used to identify and examine *GxE* interactions on BMI in the HNP_EU_ cohort of every individual variant. Here, the variable *E* represents ACEs as a whole number between zero and ten. In specific cases to validate other gene-environment interactions, *E* represents an environmental factor as defined above.

$$\mathrm{BM}I_{T}=\beta_{\text{0}}+\beta_{G}G+\beta_{E}E+\beta_{\mathrm{GxE}}\mathrm{GxE}+\beta_{1}\mathrm{age}+\beta_{2}\mathrm{sex}+\beta_{3}\mathrm{bx}+\beta_{4}\mathrm{PC}1\;+\beta_{5}\mathrm{PC}2+\beta_{6}\mathrm{PC}3+\beta_{7}\mathrm{PC}4\;+\;\beta_{8}\mathrm{PC}5+\varepsilon$$ (1)

The GWEIS was performed in *PLINK* v2.0 using a glm with the inclusion of an interaction modifier to include genotype × covariate interaction terms (Chang et al., 2015). The null hypothesis *H*_*0*_ *: β*_*GxE*_ *= 0* was tested for each of the five million high-quality variants. The covariate *bx* represents the specific bioinformatic pipeline used for variant calling. Variants of interest were examined further with lm and glm functions in **R**. Results are presented as raw *p*-values in Supplementary Table S2. A QQ plot of the results can be found in Supplementary Figure S1.

### GWAS

In the Supplementary Materials, we present a GWAS to replicate previously published genome-wide associations with BMI. A canonical linear regression equation under the assumption of the additive genetic model was used:

$$\mathrm{BM}I_{T}=\beta_{\text{0}}+\beta_{G}G\;+\;\beta_{1}\mathrm{age}+\;\beta_{2}\mathrm{sex}+\beta_{3}\mathrm{bx}+\beta_{4}\mathrm{PC}1\;+\beta_{5}\mathrm{PC}2+\beta_{6}\mathrm{PC}3+\beta_{7}\mathrm{PC}4\;+\;\beta_{8}\mathrm{PC}5+\varepsilon$$ (2)

Additionally presented in the Supplementary Materials, to measure the effect of ACEs in the presence of genetics, the same linear model was used with the inclusion of ACEs as a covariate:

$$\mathrm{BM}I_{T}=\beta_{\text{0}}+\beta_{E}E\;+\;\beta_{1}\mathrm{age}+\;\beta_{2}\mathrm{sex}+\beta_{3}\mathrm{bx}+\beta_{4}\mathrm{PC}1\;+\beta_{5}\mathrm{PC}2+\beta_{6}\mathrm{PC}3+\beta_{7}\mathrm{PC}4\;+\;\beta_{8}\mathrm{PC}5+\varepsilon$$ (3)

Both GWAS were performed using *PLINK* v2.0 (Chang et al., 2015). Models of specific variants of interest were examined with lm and glm functions in **R**. Results are presented with raw *p*-values in Supplementary Tables S3, S4, respectively. QQ plots for the results of each GWAS can be found in Supplementary Figures S2, S3.

### P-Values and Statistical Significance of GWAS Replication

All results are reported as raw *p*-values. A conventional Bonferroni family-wise error rate control for the significance level *α* = 0.05 yields a significance threshold of 1 × 10^−8^ (0.05/5,000,000) for five million tested variants. This provides a very conservative multiple testing correction guide. A second standard correction method is the Benjamini-Hochberg correction, that, for Eq. 2 (*G*-only), yields a false discovery rate (FDR) threshold of *FDR* = 4.60 × 10^−6^ (Benjamini and Hochberg, 1995). Similarly, a Benjamini-Hochberg FDR threshold for Eq. 3 (*G+E*) is 6.09 × 10^−7^ (Benjamini and Hochberg, 1995).

### Statistical Power of GWAS and GWEIS in HNP_EU_

QUANTO (Gauderman, 2002) was used to estimate sample sizes needed to attain at least 80% statistical power for a range of MAFs and main genetic effect sizes in (Eq. 2) under the additive genetic model and a two-sided Type I error at 5% significance in a standard genome-wide association analysis. Power calculations are based on normally distributed values; thus, power and effect sizes were based on BMI_T_, with *µ*_BMIT_ = 0 and σ_BMIT_ = 1. Tests of association with variants of MAF of 35% or greater were able to detect effect sizes of 0.04 BMI_T_ units with power of 83.7% using *N* = 11,880 participants (Gauderman, 2002). Tests of association with variants of MAF ≥ 3.5% detected effect sizes of 0.1 BMI_T_ units with power greater than 80% using *N* = 11,615 participants. (The effect size of 0.04 BMI_T_ units represents the change between 29.01 and 29.24 kg*/m* ^*2*^ or between 50.16 and 50.91 kg*/m* ^*2*^. Similarly, an effect size of 0.1 BMI_T_ units represents changes between raw BMI values 16.14 kg*/m* ^*2*^ and 16.31 kg*/m* ^*2*^; 24.17 kg*/m* ^*2*^ and 24.59 kg/m^2^; or 33.57 kg/m^2^ and 34.34 kg/m^2^. Examples can be seen in Supplementary Table S1). Power to detect interactive effect sizes was also performed with QUANTO. As power calculations for the effect size *β*_*GxE*_ depend on the values of *β*_*G*_ and *β*_*E*_ (Gauderman, 2002), these calculations were performed post-hoc. Sample sizes to attain at least 80% statistical power to detect *β*_*GxE*_ values for the 55 variants identified in the GWEIS are included in Supplementary Table S2, following similar analyses of Winham, Yang, and Zhao (Yang et al., 2010; Winham and Biernacka, 2013; Zhao et al., 2016). Statistical power was computed in the same manner using Quanto for the smaller cohorts in the Supplementary Materials.

## Results

### ACEs and Adult Obesity in the HNP

**FIGURE 1:**
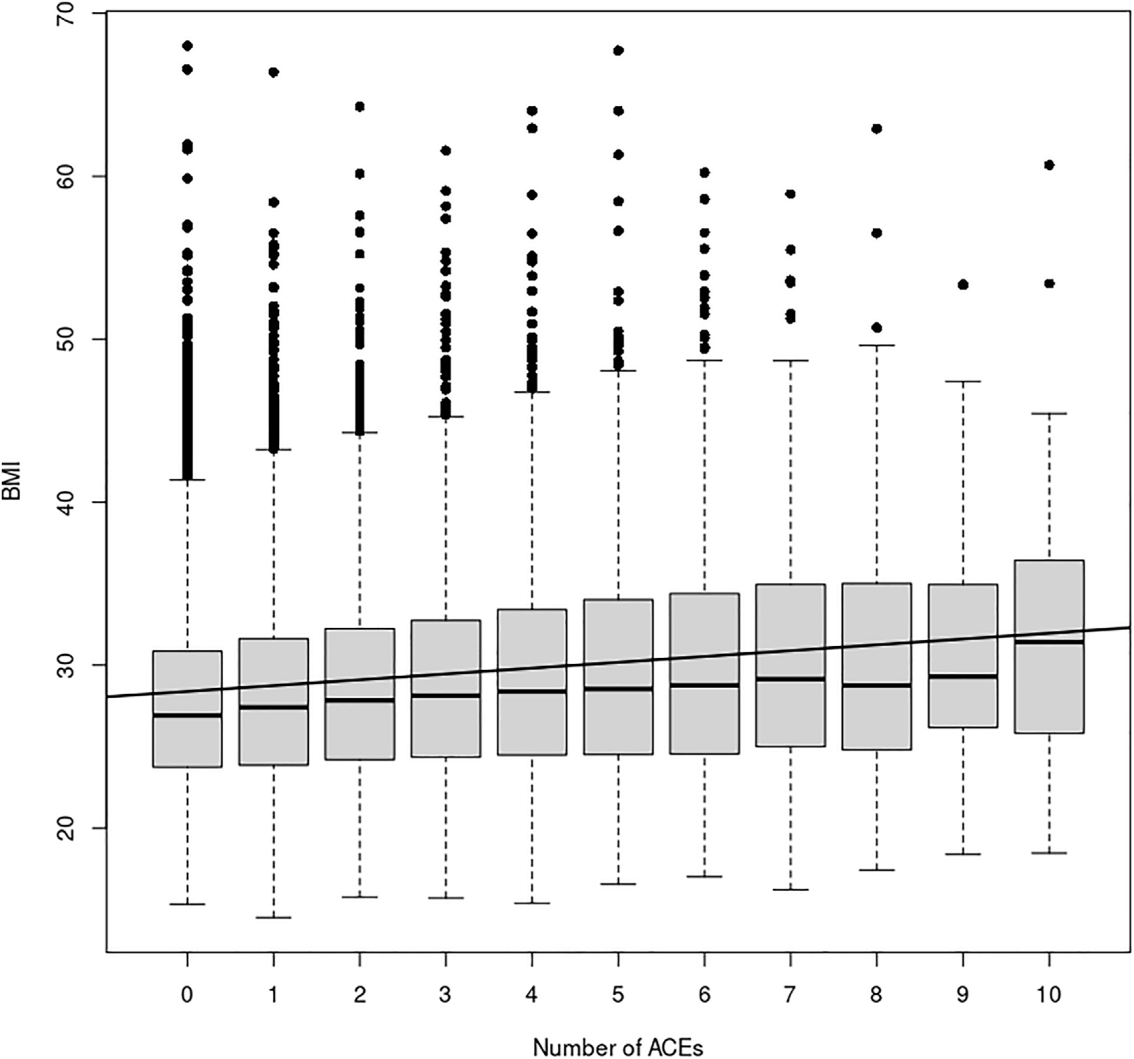
ACE Relationship vs. BMI. This figure shows the relationship between the number of ACEs experienced and average BMI in each ACEs group, irrespective of age, sex, or ethnicity. The black line depicts the simple linear regression with slope 0.36 (*p* << 2 × 10^−16^) and y-intercept 28.03. Additionally, a simply one-way ANOVA shows a statistically significant difference in the BMI index between the ACE groups (*p* < 2 × 10^−16^).

Results of the ACE questionnaire when applied to the HNP closely followed those of the original Center for Disease Control-Kaiser Permanente ACE Study (Centers for Disease Control and Prevention, 2019). The HNP survey recorded that 65.8% adults experienced at least one type of ACE in childhood, and that 24.1% have endured four or more different types of ACEs. Table 1 presents the distribution of ACEs in the 15,886 HNP participants who also have cross-referenced BMI values in the EHR; Table 2 presents the distribution for the European HNP participants. Supplementary Table S5 further breaks down this distribution by specific ethnicity. A simple linear regression using only age and sex as covariates shows that the number of ACEs (zero - ten) is a statistically significant predictor of BMI in the HNP irrespective of ethnicity (*p* << 1 × 10^−16^). With each 0.37 ACE encounter, the BMI level of HNP participants increases by 1 *kg/m* ^*2*^ unit. Including ethnicity as a covariate lowers the ACE effect to 0.35, and the *p*-value is again notably small (*p* << 1 × 10^−16^). This relationship can be seen in Figure 1 and Supplementary Table S6. Odds ratios in Table 3 and Table 4 show that participants who experienced one or more ACEs were 1.5 times more likely to become obese adults and even greater likelihood of becoming severely obese. Additionally, participants in any ethnicity with four or more ACEs were more than twice as likely to become severely obese.

**TABLE 1:** Distributions of ACEs in the HNP of all ethnicities.

Num ACEs	Mean BMI	*N*	(%)
0	27.90	5,434	34.25
1	28.44	2,999	18.90
2	28.90	2054	12.95
3	29.35	1,564	9.86
4	29.58	1,224	7.71
5	28.92	884	5.57
6	30.12	713	4.49
7	30.27	465	2.93
8	30.26	325	2.05
9	30.76	150	0.95
10	32.18	54	0.34

**TABLE 2:** Distributions of ACEs in the European HNP only.

Num ACEs	Mean BMI	*N*	(%)
0	27.86	4,539	35.35
1	28.37	2,451	19.09
2	28.75	1,652	12.87
3	29.32	1,252	9.75
4	29.53	962	7.49
5	29.78	673	5.24
6	30.23	557	4.33
7	29.97	374	2.91
8	30.43	236	1.84
9	31.10	114	0.89
10	31.70	29	0.22

**TABLE 3:** Odds ratios between the ACEs and obesity in the HNP across all ethnicities.

	≥1 ACEs	≥4 ACEs
Obese	1.52; [1.42, 1.64]	1.85; [1.69, 2.02]
Severely Obese	1.63; [1.48,1.80]	2.12; [1.90, 2.38]

Presents the odds ratios and 95% confidence intervals computed by Fisher Exact Tests between the HNP across all ethnicities.

**TABLE 4:** Odds ratios between ACEs and obesity in the European HNP.

	≥1 ACEs	≥4 ACEs
Obese	1.51; [1.40, 1.64]	1.64; [1.67, 2.03]
Severely Obese	1.62; [1.45,1.81]	2.16; [1.90, 2.46]

Presents the odds ratios and 95% confidence intervals computed by Fisher Exact Tests between Europeans in the HNP. These tables demonstrate associations between the ACE score of ≥1 and ≥4 and obese and severe obese HNP participants. ACE controls were those participants with zero ACEs. Obese controls were participants with BMI ≤ 25 kg*/m* ^*2*^).

### GxE Interactions in Europeans

**FIGURE 2:**
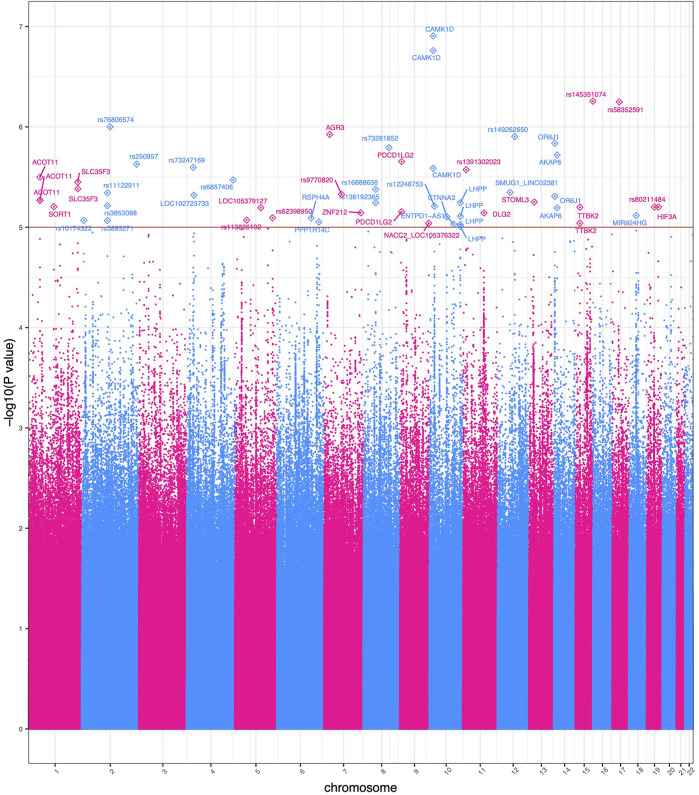
Manhattan plot of significant GWEIS results. Each point in this figure represents a result of a single variant’s genotype-environment analysis. The *x*-axis represents the genomic position of each of 4,876,698 variants. The *y*-axis represents −log_10_-transformed raw *p*-values of each genotypic association. For ease of viewing, only variants in genes above the horizontal line *α* = 1 × 10^−5^ are annotated.

Using Eq. 1 resulted in 55 variants with evidence of a genome-wide genotype-ACE interaction effect (*p* < 1 × 10^−5^). This is similar to the suggestive *p*-value threshold utilized by Wu *et al.* and others in *GxE* studies (Wu et al., 2014; Biernacka et al., 2016; Dahlin et al., 2020). Results are presented in Figure 2.

**FIGURE 3:**
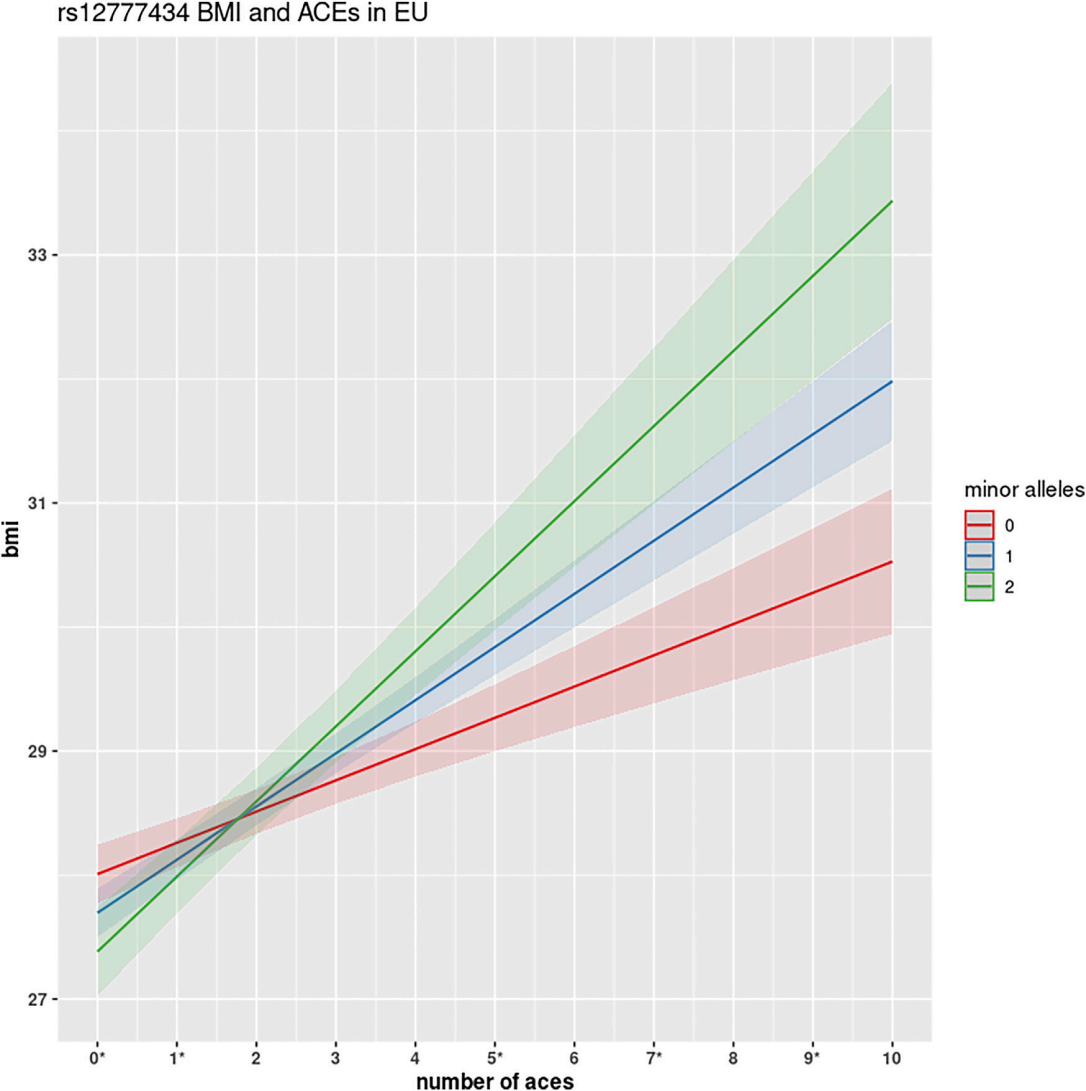
GWEIS identification of rs12777434 interaction with ACEs. This figure shows the interactive effect of variant rs12777434 in *CAMK1D.* Homozygotes in the reference allele show a substantial increase in BMI for each number of ACEs encountered (0.24 kg*/m* ^*2*^). However, with each copy of the minor allele, participants show much greater changes: 0.48 kg*/m* ^*2*^ and 0.57 kg*/m* ^*2*^, respectively. Differences in BMI between the three genotypes are shown to be statistically significant at ACEs *N* = 0, 5, 9 at significance level *α* = 0.05, and at *N* = 6 and 7 ACEs at significance level *α* = 0.10, using simple one-way ANOVA analysis.

Two variants rs12777434 (*p*-value = 1.24 × 10^−7^) and rs71477259 (*p*-value = 1.73 × 10^−7^) in *CAMK1D* showed the strongest *GxE* association. BMI levels of individuals with one or two copies of the minor allele are affected (positively) at a notably greater rate with increase in ACE events than those with zero copies of the minor allele as shown in Figure 3. The rate of increase is 0.24 kg*/m* ^*2*^ with each adverse childhood encounter in a participant with no minor alleles; 0.48 kg*/m* ^*2*^ per ACE with one minor allele; 0.57 kg*/m* ^*2*^ with two minor alleles. Thus, the rate of BMI increase across ACEs doubles in heterozygotes. Interestingly, the minor allele of rs12777434 in *CAM1KD* is also statistically significantly associated with notably higher BMI levels in HNP_EU_ Never Drinkers (*p* = 0.03), whereas the variant has no effect on BMI in any other group of drinkers. These interactions are shown in Supplementary Figure S4.

Another interactive effect is observed by variant rs62398950 (*p*-value = 8.05 × 10^−6^) in a non-coding region on chromosome 5. Alone, its protective effect (−0.51 kg*/m* ^*2*^ for each copy of the minor allele), is not significant (*p* = 0.41). When considered with the covariate ACEs, its effect size does not change, nor does the *p*-value of the hypothesis test for the *β*_*G*_ coefficient. However, when examining the interaction of this variant with ACEs, the protective effect of the variant becomes clear in the minor allele carriers (MAF = 0.84%). The difference in slopes between individuals with one minor allele and those with no minor alleles is 1.24 kg*/m* ^*2*^: thus minor allele carriers will, in general, have 1.24 BMI points less per each number of ACE endured than those with no minor allele (Supplementary Figure S5). The minor allele shows a notable and statistically significant protective effect against obesity in HNP_EU_ participants with greater ACEs endured in childhood.

Additionally, variant rs76806574 (*p*-value = 9.98 × 10^−7^) in a non-coding region on chromosome 2, displays a similar protective effect. The most notable differences and statistically significant differences are for participants with *N* = 8 ACEs, where BMI differences between the participants carrying no alternative allele and one alternative allele is 5.7 BMI index points, and this difference is significant at the α = 0.05 level. The variant rs142517237 in *HIF31* shows one of the greatest interactive effects: although it has no previous link to BMI, its minor allele carriers show a *decrease* of 2.9 kg*/m* ^*2*^ for each ACE encountered, compared to an *increase* of 0.36 kg*/m* ^*2*^ for its non-carriers.

Variant rs145351074 (*p*-value = 5.55 × 10^−7^) on chromosome 15 also showed prominent differences in effects based on genotype. It is slightly upstream of the gene *LOC105376730*, which produces a long non-coding RNA (lncRNA). For participants with no alternative alleles, BMI increases slowly with the number of reported ACEs. However, for those participants with one or more alternative alleles, BMI levels increase at a much faster rate, similar to what is seen in Figure 3.

### GxE Results of Smaller Cohorts

The GWEIS results presented in this manuscript focus on HNP_EU_ participants due to its large cohort size. As we recognize the importance of multi-ethnic studies, we also examine variants with suggestive evidence of interaction in both the HNP_AA_ cohort (Supplementary Figure S6) and the HNP_LX_ (Supplementary Figure S7) cohorts, both of which have notably smaller sample sizes. Although underpowered statistically, we observed variants of interest, such as rs544101 and rs8004002, that presented suggestive evidence of interaction not only in the HNP_EU_, but also in the HNP_AA_ and HNP_LX_ cohorts, respectively. Both of these variants associate with different rates of BMI across genotypes and across the number of ACEs in all three ethnic cohorts. Furthermore, PhenoScanner demonstrates that rs8004002 is found in a known schizophrenia-related gene similar to European GWEIS results (Staley et al., 2016; Kamat et al., 2019). Additional results from the smaller cohorts are presented in the Supplementary Material.

### Replication of Previously Published BMI Associations

To the best of our knowledge, this study is unique in its genome-wide examination of gene-environment effects of ACEs and BMI. For this reason, we validate our approach based on current published BMI GWAS studies. We perform a GWAS (Eq. 2) on the HNP_EU_ to replicate the variant and gene effects that have previously been published (Supplemental Material; Supplementary Table S3). Additionally, we use Eq. 3 and the HNP_EU_ cohort to examine associations with BMI including ACEs as a covariate to examine *G+E* associations. (Supplemental Material; Supplementary Table S4; Supplementary Figure S8). The GWAS results using Eq. 2 show that we replicate many of the expected canonical BMI associations (Supplemental Material). Further, using Eq. 3, which includes ACEs as a main effect covariate, we observed that all significant associations fall solely within the FTO gene (Supplemental Material).

### GxE Validation With Alternative Environmental Exposures

Several other published BMI interactions, based on different environmental factors, were also replicated in order to provide additional evidence that the HNP_EU_ cohort and our methods are suitable for determining the interactive effects between genotype and ACEs on BMI. We found that the *FTO* variant rs1558902 exhibited a significant interaction effect with alcohol intake to modify BMI levels, similar to previous observations (Young et al., 2016; Rask-Andersen et al., 2017). Moreover, both rs9939609 and rs10517309 produced significant interaction effects on BMI using education level and cigarette smoking respectively (Freathy et al., 2011; Corella et al., 2012).

## Discussion

Standard GWEIS analyses often target a handful of variants that are known to have a genetic effect on the phenotype of interest. Here, we perform a genotype-environment interaction analysis on each of the five million variants on our HNP exome platform to capture all interaction effects that might not be identified in a standard targeted analysis. An interaction of statistical significance implies that a participant having experienced ACEs carrying a minor allele of a variant reacts differently with respect to BMI levels than an individual not carrying the minor allele. For example, in certain cases, a minor allele of a variant may protect an adult who experienced ACEs from increased BMI, whereas the reference allele will increase the likelihood of that person experiencing increased BMI levels. In other cases, the opposite trend may occur, in which the minor allele could increase the risk of elevated BMI for someone who has experienced an ACE.

### ACEs and Adult Obesity in the HNP

Here we study a multi-ethnic cohort of notable size (15,866) and first observe its incidence of ACEs: 65.7% of participants have experienced at least one type of adverse event in childhood, and 24.0% have experienced four or more ACEs. Although study populations are difficult to compare, based on different socio-demographic characteristics, we do see a greater incidence in ACEs in the HNP than in other cohorts. For example, the Behavioral Risk Factor Surveillance System (BRFSS) is a telephone-based survey of adults during 2015–2017 across 27 states that includes ACE questions with responses of 144,000 adults (Merrick et al., 2019). The study reported 60.9% of its adults had experienced at least one and 15.6% had endured more than four ACEs in their lifetime (Merrick et al., 2019; Jones et al., 2020). Felitti’s original 1998 study of 8,056 individuals reported that 50% of its participants had experienced at least one ACE; Godoy reported that at least 50% of US adults had experienced one type or more of ACEs (Jones et al., 2020; Godoy et al., 2021).

Danese presents no less than 38 studies that examine possible links between childhood adversity and obesity, again, with a variety of demographics (Danese and Tan, 2014). Here, we show that a multi-ethnic cohort with a high rate of some college education (88%) and a higher-than-average median income ($72,303) shows a definitive association between the number of different ACEs experienced in childhood and adulthood BMI. It has been previously determined that lower educational level and lower income are both linked to higher BMI and a greater number of ACEs (Hajian-Tilaki and Heidari, 2010; Hermann et al., 2011; Cohen et al., 2013; Ogden et al., 2017; Hardcastle et al., 2018; Blair et al., 2019; Giano et al., 2021; Hansen et al., 2021; Haugland et al., 2021), which suggests that we may be underestimating the factors that impact BMI in the HNP. Via linear regression, we show that each 0.37 ACE experienced increases adult BMI levels by 1 *kg/m* ^*2*^. Odds ratios showed first that HNP participants experiencing one or more ACEs were 1.5 times more likely to become obese adults and an even greater likelihood of becoming severely obese. In comparison to the review of Wiss in 2020, the trend in the HNP is greater (Wiss and Brewerton, 2020). Additionally, HNP participants of any ethnicity with four or more ACEs were 2.16 times more likely to become severely obese; again, in comparison with other studies, this trend is also higher. Anda’s study (75% European cohort), reported a ratio of 1.9 in this same comparison (Anda et al., 2006), and Fuemmeler reported a two-fold odds ratio (Fuemmeler et al., 2009).

### GWEIS of HNP_EU_

The GWEIS identified 55 variants with strong interactive effects. It is of interest that none of these variants lie in the *FTO* gene, nor on chromosome 16. Many of our GWEIS findings have similar outcomes as those in the study by Young *et al.*, in which the effects of *FTO* on BMI are diminished by environmental variables. In Young’s study, the variables alcohol consumption, sleep duration, and overall diet over-rode and decreased the canonical effect of *FTO* variants on BMI (Young et al., 2016). Here we see a similar phenomenon with the ACE environmental variable; genetic variants alone may have little or no effect on BMI, but when paired with an environmental variable, their effects are notable and significant.

The variant rs12777434 exhibited the most significant interactive effect, yet the variant alone did not associate with levels of BMI and would have been missed in a conventional GWAS (*p* = 0.92, with an effect size of 0.01 kg*/m* ^*2*^). In combination with the environmental effect, however, the variant significantly and notably increased the BMI levels with each ACE endured, indicating that the genetic effect on BMI of this variant is modified by the number of ACEs encountered. This variant is in *CAMK1D,* a gene which codes for a Calcium/calmodulin-dependent protein kinase and has broad expression in the brain according to the protein atlas (Uhlén et al., 2017). *CAMK1D* is associated with schizophrenia according to the Neale lab and PhenoScanner [http://www.nealelab.is/uk-biobank/] (Staley et al., 2016; Kamat et al., 2019). The association to schizophrenia is of interest, as it may play a part in some of the association between BMI and ACEs: participants with schizophrenic symptoms in study by Prokopez *et al.* were more likely to have experienced an ACE, and those with multiple ACEs have poorer overall outcomes and more severe schizophrenic symptomatology (Vallejos et al., 2017; Prokopez et al., 2020).

Indeed, the HNP follows these trends as well. When compared to clinical controls (with exclusionary criteria incorporated), we see an extraordinary relationship between ACEs and schizophrenia: individuals with schizophrenia are 18.5-fold more likely to have experienced one or more ACEs in their childhood (*p* < 1 × 10^−10^), and participants with schizophrenia are 31.7 times more likely to have experienced four or more ACEs (*p* < 1 × 10^−14^). HNP patients with schizophrenia have an average BMI of 31.3 kg*/m* ^*2*^, while their controls have a mean BMI of 28.8 kg*/m* ^*2*^ *.*

The direct molecular mechanism through which rs12777434 acts on BMI and schizophrenia is currently unclear; however, variants within *CAMK1D* have been shown to affect its gene expression, leading to altered glucose processing through decreased gluconeogenesis and increased glycogen storage. Increased glycogen storage will directly alter BMI as obesity is associated with larger glycogen stores in adipose tissue (Ceperuelo-Mallafré et al., 2016). Additionally, increasing evidence shows that altered glucose processing is an early biomarker in schizophrenic patients as it can affect signaling and dysregulate astrocyte-neuron compartments (Zhang et al., 2015; Roosterman and Cottrell, 2021). Furthermore, patients suffering from schizophrenia may have additional triggers on their BMI. It has been established that participants diagnosed with schizophrenia have notably increased levels of BMI due to SDOH including lower socioeconomic status, lower education level, lack of access to healthcare, and lower physical activity, in addition to the effect of antipsychotic drugs (Coodin, 2001; Annamalai et al., 2017).

Additional points of evidence linking schizophrenia and BMI in HNP_EU_ were observed in variants within eleven genes: *SORT1*, *SLC35F3*, *ZNP212*, *PDCD1LG2*, *CTNNA3*, *LHPP*, *DLG2*, *SMUG1*, *STOML3*, *AKAP6*, *HIF3A* (Buttenschøn et al., 2015; Corvin, 2010; Gardiner et al., 2013; Grove et al., 2014; Li et al., 2016; 2017; MacLaren et al., 2011; Neff et al., 2009; O'Connell and Coombes, 2021; Shao and Vawter, 2008; Smeland et al., 2017; Thippeswamy and Davies, 2021). A complete analysis of the molecular mechanisms by which the variants in these genes influence schizophrenia and BMI is outside the scope of this paper; we briefly highlight a few variants in genes previously linked to schizophrenia. We observed four variants (rs71026101, rs36096707, rs11245311, rs12771611) within the schizophrenia-related *LHPP* gene [http://www.nealelab.is/uk-biobank/] (Staley et al., 2016; Kamat et al., 2019) that presented evidence of significant interactive effects (Supplementary Table S2). *LHPP* shows broad expression in the brain and could alter cellular signal transduction in patients suffering from major depressive disorder (MDD); although, its exact function has yet to be elucidated (Neff et al., 2009). Furthermore rs77744003, a variant within the schizophrenia-related *STOML3* gene, showed an interactive effect. Similar to *LHPP*, this gene has broad expression in the brain, including the hypothalamus and hippocampus, and functions to maintain acid-sensing cation channels by the binding of cholesterol (Conrard and Tyteca, 2019; Thippeswamy and Davies, 2021). In addition to schizophrenia, the genomic region around *STOML3* has been associated with autism spectrum disorders as well as psychotic depression (Thippeswamy and Davies, 2021).

The interaction between genetics, schizophrenia, and ACEs highlights the complex and multifactorial interactions that drive common traits such as BMI. For example, a recent BMI study with approximately 700,000 individuals observed that 750 BMI-associated variants accounted for only approximately 6% of variation in BMI (Yengo et al., 2018; Loos and Yeo, 2021). This shows that a large amount of BMI variability is still unaccounted for using standard genetic analysis. However, the observed BMI interaction effects in this study imply that history of life events and disease state are also important in the genetic associations with BMI, and could identify new targets as well as uncover some of the missing variability of these complex traits for those who have experienced childhood maltreatment in the past.

Two other variants demonstrating significant *GxE* effects with BMI include rs145351074 and rs76806574. These variants are located in *LOC105376730* on chromosome 15 and *ENSG00000286481* on chromosome 2, respectively, and code for long non-coding RNA (lncRNAs). Again, neither variant is a predictor of BMI when considered as a main effect (*p* = 0.42 and *p* = 0.84, respectively). Previous research found that the expression of several lncRNAs become dysregulated in obese patients (Sun et al., 2016). Furthermore, other cell and animal studies on lncRNAs show that they respond to external environmental exposures and stimuli, such as ultraviolet radiation, smoke, and chemical exposures (Zhou et al., 2015; Karlsson and Baccarelli, 2016; Prokopez et al., 2020). We hypothesize that one or more ACEs could present an external stimulus that possibly overrides the effect of these variants, and those with the alternative allele may undergo dysregulation of this lncRNA. This dysregulation may be what causes a notable decrease in the BMI for these participants carrying the minor allele, but only for those who have also experienced at least one type of ACE.

### Validation of GxE With Alternative Environmental Factors

Studies of the HNP_EU_ previously used the United Kingdom Biobank (UKBB) as a validation cohort (Cirulli et al., 2020; Read et al., 2021). There was a lack of consistency between the childhood trauma questions in the HNP study and the UKBB “Traumatic event questions” (Field IDs 20487, 20489, 20489, 20490, 20491): only two of the ten standard ACE questions were similar to the UKBB’s five questions. Thus, the UKBB cohort was inappropriate for direct GWEIS replication. To evaluate our methods and interaction model, we replicated several other BMI interaction effects using alternative environmental exposures. For example, both Young and Rask-Andersen observed an interaction effect between alcohol and *FTO* variant rs1558902 in the predominantly European UKBB (Young et al., 2016; Rask-Andersen et al., 2017). In both these studies, alcohol intake modified the effect of the *FTO* variant on BMI: the effect of the minor allele on participants who drank more frequently was much smaller than those who drank less frequently. This result was replicated in the HNP_EU_ (*p* = 0.012) and is most notable in the “Never” alcohol consumption group (Supplementary Figure S9). Without the consideration of alcohol intake, each copy of the minor allele raises the HNP_EU_ BMI by 0.66 kg*/m* ^*2*^ in the HNP. When considering this effect across alcohol consumption groups, the effect of the genotype changes: in the Never drinker category, one copy of the minor allele is associated with changes in BMI levels of 1.19 kg*/m* ^*2*^, whereas two copies are associated with an increase of 1.97 kg*/m* ^*2*^. Similarly, genotype effects in the “>4/Week” group were much less: differences in BMI between zero and one copies of the minor allele were 0.61 kg*/m* ^*2*^, and differences between one and two copies of the minor allele were 0.36 kg*/m* ^*2*^

Furthermore, previous research has established that educational levels are related to BMI (Corella et al., 2012). Interactions between rs9939609 and educational levels were examined by Corella in a modestly sized Mediterranean cohort in 2015 (Corella et al., 2012). When grouping participants into those with university training and those without, the interactive effect was trending towards significance (Corella *p* = 0.048; HNP_EU_ *p* = 0.092), where the minor allele had a much greater positive effect on individuals without university training (Corella et al., 2012). Another environmental factor that is often studied in tandem with obesity is cigarette smoking. Previously, Freathy and Taylor examined the *GxE* effect of the variant rs10517309 with smoking and BMI levels in European populations (Freathy et al., 2011). The effect is notable in the non-smokers in both studies, but not in the smokers. This behavior was also found in the HNP_EU_ cohort: the minor allele of rs10517309 was associated with greater BMI in non-smokers and lower BMI in smokers, which is shown in Supplementary Figure S10.

### Limitations of the Study

One clear limitation of this study is the sample size of smaller ethnic cohorts in the HNP. As such, the genetic examinations that are provided in the Supplementary Material should be replicated when the cohort is better populated with larger ethnic subcohorts. Further, although this study accounted for all ten standard ACE questions, we computed and used only one agglomerative score in the GWAS and GWEIS investigations. It is possible that if environmental exposure were represented differently, such as a case vs. control model, the findings may be observed differently. This type of validation would provide a basis for a relevant follow-up study. Another limitation is that the number of ACE instances each participant encountered was not recorded. We have determined this is true of all retrospective ACE studies. A final issue in this study, as well as all retrospective studies, is that recollection of childhood events may not always be completely accurate.

## Conclusion

ACEs have a profound impact on adult health and traits that impact health outcomes such as BMI and schizophrenia. The relationship between retrospectively recalled ACEs and adult BMI in the HNP was shown to be strongly significant with a notably large positive effect size. BMI is a well-studied phenotype in many standard genome-wide approaches, yet its gene-environment associations are far less known. The GWEIS performed here identified several unknown interactive effects based on childhood adverse events. Surprisingly, a number of the variants that resulted in interesting interactive effects were not, by themselves, predictors of BMI levels. In simple terms, this demonstrates that many conventional genes linked to BMI (e.g., *FTO*) have less impact on BMI when paired with the incidence of ACEs. Studies able to include environmental or SDOH variables when examining complex traits may account for some missing genetic heritability for those who are exposed to the environmental factor of interest.

This unique examination highlights several of the interactive effects between genetics and behavioral life experiences, and the consequences thereof on population health. Particularly, this study shows that the largely preventable negative health impacts of ACEs modulate purely genetic associations to an often detrimental effect on health. Simply stated, poor health outcomes result from lifestyle-driven events, and these health outcomes increase notably with specific genetic mutations. Conversely, a number of variants have already been shown to play a strong role in the increase of unhealthy BMI levels; when considered in tandem with environmental events such as ACEs, these effects can multiply in strength, resulting in a much worse state of disease. Thus, future emphasis in large population health studies must be placed on the strongly negative impact of adverse events encountered in childhood and the interactive effects of these events with specific genetic variations. Considering a patient’s social environment such as adverse experiences in childhood will provide a more complete clinical arsenal for overall better patient health.

## Data Availability

The data analyzed in this study are subject to the following licenses/restrictions: These data are available to qualified researchers upon reasonable request and with permission of the Center for Genomic Medicine and Helix. Researchers who would like to obtain the raw genotype data related to this study will be presented with a data user agreement which requires that no participants will be reidentified and no data will be shared between individuals or uploaded onto public domains. Due to the public nature of this article, and genetic privacy requirements, the Center for Genomic Medicine, and Helix require that the summary statistics of only 10,000 variants be made publicly available. This is the amount of data considered to be insufficient to enable a re-identification attack. The summary results of the most statistically significant 10,000 variants in this study will be made available on https://datadryad.org/. We attest that one author had full access to all the data in this study and takes responsibility for its integrity and the data analysis. The Center for Genomic Medicine encourages and collaborates with scientific researchers on an individual basis. Examples of restrictions that will be considered in requests to data access include but are not limited to: 1. Whether the request comes from an academic institution in good standing and will collaborate with our team to protect the privacy of the participants and the security of the data requested 2. Type and amount of data requested. 3. Feasibility of the research suggested. 4. Amount of resource allocation for the IHI and Renown Hospital required to support the collaboration Any correspondence and data availability requests should be addressed to Joseph Grzymski at (Joe.Grzymski@dri.edu) or Craig Kugler (Craig.Kugler@dri.edu). EHR Data: EHR data for the Healthy Nevada Project cohort are subject to HIPAA and other privacy and compliance restrictions. The mean quality-controlled BMI for each individual de-identified participant will be made available on https://datadryad.org/.
