# Supplementary material for: The Impact of ACEs on BMI: An Investigation of the Genotype-Environment Effects of BMI
Source: Front Genet. 2022 Mar 7;13:816660. doi: 10.3389/fgene.2022.816660 (PMC8942770; doi:10.3389/fgene.2022.816660)
Supplement: Supplementary file 3 [file DataSheet1.PDF]

Supplemental Material

Results

Interacting Variants with ACEs Identified by GWEIS in LatinX
Although the HNP_{LX} cohort was too small to conduct statistically powerful interactive studies (N=1,774), we did examine a number of the variants from Supplementary Table S2 in this cohort. Variants rs149262650 on chromosome 12, rs77744003 in *STOML3*, and rs8004002 in *AKAP6* in Supplementary Table S2 also exhibited significant interactive effects in this LatinX cohort as well as the HNP_{EU}.

Interacting Variants Identified with ACEs by GWEIS in African Americans
The variants rs115847516, rs11206385 (Supplementary Figure S6) and rs544101 in *ACOT11* all presented with significant interactive mechanisms at $p \leq 0.01$ in the African American cohort (HNP_{AA}). Where the minor allele in rs115847516 and rs11206385 is protective, the minor allele in rs544101 associates with higher BMI with increasing number of ACEs.

Gene-only GWAS
These results align with many reported BMI associations and include variants in the *FTO, NEGR1*, *BDNF*, *ADCY3*, near and in *SEC16B*, near *TMEM18,* and near *MC4R* (Akbari et al., 2021; Chalazan et al., 2021; Frayling et al., 2007; Graff et al., 2013; Iepsen et al., 2018; Loos et al., 2008; Namjou et al., 2013; Rask-Andersen et al., 2017; Sahibdeen et al., 2018; Schlauch et al., 2019; 2020; Scuteri et al., 2007; Song et al., 2008; Speliotes et al., 2010; Thorleifsson et al., 2009; Willer et al., 2009) (Supplementary Table S3). Two variants in *LINC01648*, rs1498244 and rs452452, not previously linked to BMI showed pronounced effect sizes that translate to an increase in BMI of 9.6 and 9.9 kg/m^2, respectively, per copy of the minor allele. Further, the variant rs150097123, in *LINC01684*, shows an increase of 1.24 kg/m^2 per copy of the minor allele. Neither this variant nor gene have been linked to BMI to our knowledge. The low frequency variant rs57803073 (MAF=1.5%) in *LOC105372385*, and near to *CAPNS1* also has a notable main effect on BMI that translates into 1.11 kg/m^2 in the HNP_{EU}. Although not implicated in direct BMI associations, the *CAPNS1* gene has been linked to liver cancer, hypertensive heart disease, and high triglycerides in the UK Biobank (Kamat et al., 2019; Staley et al., 2016).

G+E GWAS
All 38 significant variants are in the *FTO* gene (Supplementary Table S4; Supplementary Figure S7). Note that two variants, rs1861866 and rs10852521, are protective against increased BMI and obesity, independent of ACEs in the HNP_{EU}. These two variants are in high LD with one another, but not with the other significant variants in *FTO*. Their protective effect sizes are similar to those in the UK BioBank and others (Kamat et al., 2019; Staley et al., 2016). Interestingly, the number of ACEs was a significant predictor of BMI in 99.89% of the 5 million models at the Bonferroni significance level $1x10^{-8}$. This indicates that the number of ACEs is a significant driver of BMI in the HNP_{EU}.

Discussion

Gene-only GWAS

Since the first published association of *FTO* with BMI in 2007, variants in *FTO* consistently link to BMI and obesity across ethnicities (Akbari et al., 2021; Chalazan et al., 2021; Fawcett and Barroso, 2010; Frayling et al., 2007; Graff et al., 2013; Iepsen et al., 2018; Loos et al., 2008; Namjou et al., 2013; Rask-Andersen et al., 2017; Sahibdeen et al., 2018; Schlauch et al., 2019; 2020; Scuteri et al., 2007; Song et al., 2008; Speliotes et al., 2010; Thorleifsson et al., 2009; Willer et al., 2009; Young et al., 2016). Thus, the results of the standard *G*-only GWAS (Equation 2) were not surprising: they included many *FTO* results and followed many previously reported variants and genes (Akbari et al., 2021; Chalazan et al., 2021; Frayling et al., 2007; Graff et al., 2013; Iepsen et al., 2018; Loos et al., 2008; Namjou et al., 2013; Rask-Andersen et al., 2017; Sahibdeen et al., 2018; Schlauch et al., 2019; 2020; Scuteri et al., 2007; Song et al., 2008; Speliotes et al., 2010; Thorleifsson et al., 2009; Willer et al., 2009) (Supplementary Table S3).

G+E GWAS

Results of Equation 3 yield significant associations with variants in only the *FTO* gene (Supplementary Table S4). These results support the notion that genetic effects on BMI are somewhat muted upon consideration of the number of ACEs. Several studies have suggested that *FTO* variants have off-gene effects and may be associated with BMI and obesity by regulating the expression of nearby genes (Claussnitzer et al., 2015; Jowett et al., 2010; Lan et al., 2020; Tung et al., 2014). As is well-known, variants in *FTO* are by far the most robust genetic predictors of BMI, thus their dominance in this model (*G*+*E*) is not surprising.

References

Akbari, P., Gilani, A., Sosina, O., Kosmicki, J. A., Khrimian, L., Fang, Y.-Y., et al. (2021). Sequencing of 640,000 exomes identifies GPR75 variants associated with protection from obesity. *Science* 373. doi:10.1126/science.abf8683.

Chalazan, B., Palm, D., Sridhar, A., Lee, C., Argos, M., Daviglus, M., et al. (2021). Common genetic variants associated with obesity in an African-American and Hispanic/Latino population. *PLoS ONE* 16, e0250697. doi:10.1371/journal.pone.0250697.

Claussnitzer, M., Dankel, S. N., Kim, K.-H., Quon, G., Meuleman, W., Haugen, C., et al. (2015). FTO Obesity Variant Circuitry and Adipocyte Browning in Humans. *N Engl J Med* 373, 895–907. doi:10.1056/NEJMoa1502214.

Fawcett, K. A., and Barroso, I. (2010). The genetics of obesity: FTO leads the way. *Trends Genet.* 26, 266–274. doi:10.1016/j.tig.2010.02.006.

Frayling, T. M., Timpson, N. J., Weedon, M. N., Zeggini, E., Freathy, R. M., Lindgren, C. M., et al. (2007). A common variant in the FTO gene is associated with body mass index and predisposes to childhood and adult obesity. *Science* 316, 889–894. doi:10.1126/science.1141634.

Graff, M., ndez-Rhodes, L. F. A., Liu, S., Carlson, C., Wassertheil-Smoller, S., Neuhouser, M., et al. (2013). Generalization of adiposity genetic loci to US Hispanic women. *Nutr Diabetes* 3, e85–10. doi:10.1038/nutd.2013.26.

Iepsen, E. W., Zhang, J., Thomsen, H. S., Hansen, E. L., Hollensted, M., Madsbad, S., et al. (2018). Patients with Obesity Caused by Melanocortin-4 Receptor Mutations Can Be Treated with a Glucagon-like Peptide-1 Receptor Agonist. *Cell Metabolism* 28, 23–32.e3. doi:10.1016/j.cmet.2018.05.008.

Jowett, J. B. M., Curran, J. E., Johnson, M. P., Carless, M. A., Göring, H. H. H., Dyer, T. D., et al. (2010). Genetic variation at the FTO locus influences RBL2 gene expression. *Diabetes* 59, 726–732. doi:10.2337/db09-1277.

Kamat, M. A., Blackshaw, J. A., Young, R., Surendran, P., Burgess, S., Danesh, J., et al. (2019). PhenoScanner V2: an expanded tool for searching human genotype–phenotype associations. *Bioinformatics* 35, 4851–4853. doi:10.1093/bioinformatics/btz469.

Lan, N., Lu, Y., Zhang, Y., Pu, S., Xi, H., Nie, X., et al. (2020). FTO - A Common Genetic Basis for Obesity and Cancer. *Front. Genet.* 11, 559138. doi:10.3389/fgene.2020.559138.

Loos, R. J. F., Lindgren, C. M., Li, S., Wheeler, E., Zhao, J. H., Prokopenko, I., et al. (2008). Common variants near MC4R are associated with fat mass, weight and risk of obesity. *Nat Genet* 40, 768–775. doi:10.1038/ng.140.

Namjou, B., Keddache, M., Marsolo, K., Wagner, M., Lingren, T., Cobb, B., et al. (2013). EMR-linked GWAS study: investigation of variation landscape of loci for body mass index in children. *Front. Genet.* 4, 268. doi:10.3389/fgene.2013.00268.

Rask-Andersen, M., Karlsson, T., Ek, W. E., and Johansson, Å. (2017). Gene-environment interaction study for BMI reveals interactions between genetic factors and physical activity, alcohol consumption and socioeconomic status. *PLoS Genet.* 13, e1006977. doi:10.1371/journal.pgen.1006977.

Sahibdeen, V., Crowther, N. J., Soodyall, H., Hendry, L. M., Munthali, R. J., Hazelhurst, S., et al. (2018). Genetic variants in SEC16B are associated with body composition in black South Africans. *Nutr Diabetes* 8, 43–10. doi:10.1038/s41387-018-0050-0.

Schlauch, K. A., Kulick, D., Subramanian, K., De Meirleir, K. L., Palotás, A., and Lombardi, V. C. (2019). Single-nucleotide polymorphisms in a cohort of significantly obese women without cardiometabolic diseases. *Int J Obes (Lond)* 43, 253–262. doi:10.1038/s41366-018-0181-3.

Schlauch, K. A., Read, R. W., Lombardi, V. C., Elhanan, G., Metcalf, W. J., Slonim, A. D., et al. (2020). A Comprehensive Genome-Wide and Phenome-Wide Examination of BMI and Obesity in a Northern Nevadan Cohort. *G3* 10, 645–664. doi:10.1534/g3.119.400910.

Scuteri, A., Sanna, S., Chen, W.-M., Uda, M., Albai, G., Strait, J., et al. (2007). Genome-Wide Association Scan Shows Genetic Variants in the FTO Gene Are Associated with Obesity-Related Traits. *PLoS Genet.* 3, e115–11. doi:10.1371/journal.pgen.0030115.

Song, Y., You, N.-C., Hsu, Y.-H., Howard, B. V., Langer, R. D., Manson, J. E., et al. (2008). FTO polymorphisms are associated with obesity but not diabetes risk in postmenopausal women. *Obesity* 16, 2472–2480. doi:10.1038/oby.2008.408.

Speliotes, E. K., Willer, C. J., Berndt, S. I., Monda, K. L., Thorleifsson, G., Jackson, A. U., et al. (2010). Association analyses of 249,796 individuals reveal 18 new loci associated with body mass index. *Nat Genet* 42, 937–948. doi:10.1038/ng.686.

Staley, J. R., Blackshaw, J., Kamat, M. A., Ellis, S., Surendran, P., Sun, B. B., et al. (2016). PhenoScanner: a database of human genotype-phenotype associations. *Bioinformatics* 32, 3207–3209. doi:10.1093/bioinformatics/btw373.

Thorleifsson, G., Walters, G. B., Gudbjartsson, D. F., Steinthorsdottir, V., Sulem, P., Helgadottir, A., et al. (2009). Genome-wide association yields new sequence variants at seven loci that associate with measures of obesity. *Nat Genet* 41, 18–24. doi:10.1038/ng.274.

Tung, Y. C. L., Yeo, G. S. H., O'Rahilly, S., and Coll, A. P. (2014). Obesity and FTO: Changing Focus at a Complex Locus. *Cell Metabolism* 20, 710–718. doi:10.1016/j.cmet.2014.09.010.

Willer, C. J., Speliotes, E. K., Loos, R. J. F., Li, S., Lindgren, C. M., Heid, I. M., et al. (2009). Six new loci associated with body mass index highlight a neuronal influence on body weight regulation. *Nat Genet* 41, 25–34. doi:10.1038/ng.287.

Young, A. I., Wauthier, F., and Donnelly, P. (2016). Multiple novel gene-by-environment interactions modify the effect of FTO variants on body mass index. *Nat Commun* 7, 12724–12. doi:10.1038/ncomms12724.

Figure S1

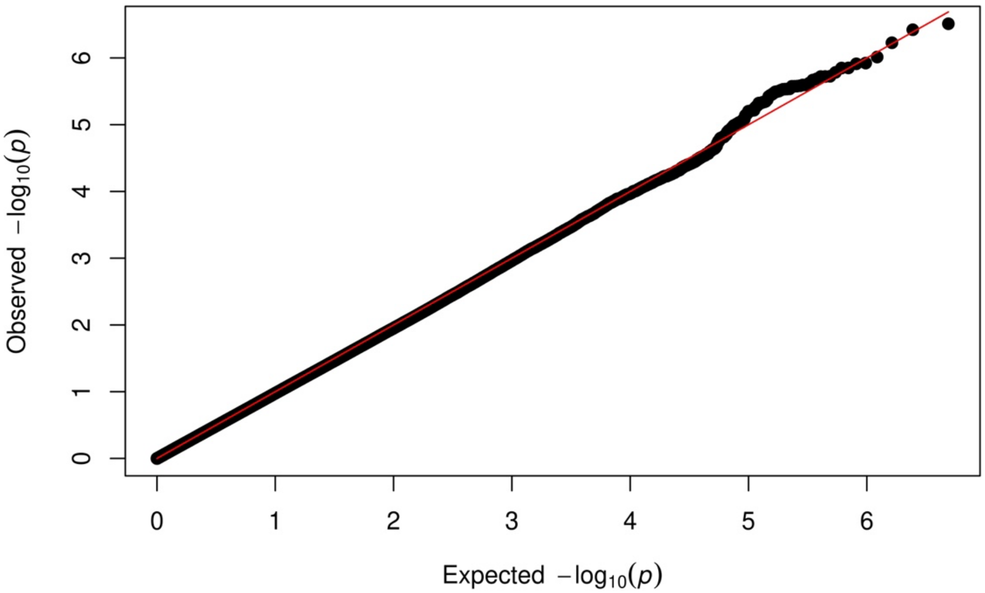

Figure S1. QQ Plot of GWEIS results. This figure shows the QQ plot for the genome-wide interaction results from Equation (1).

Figure S2

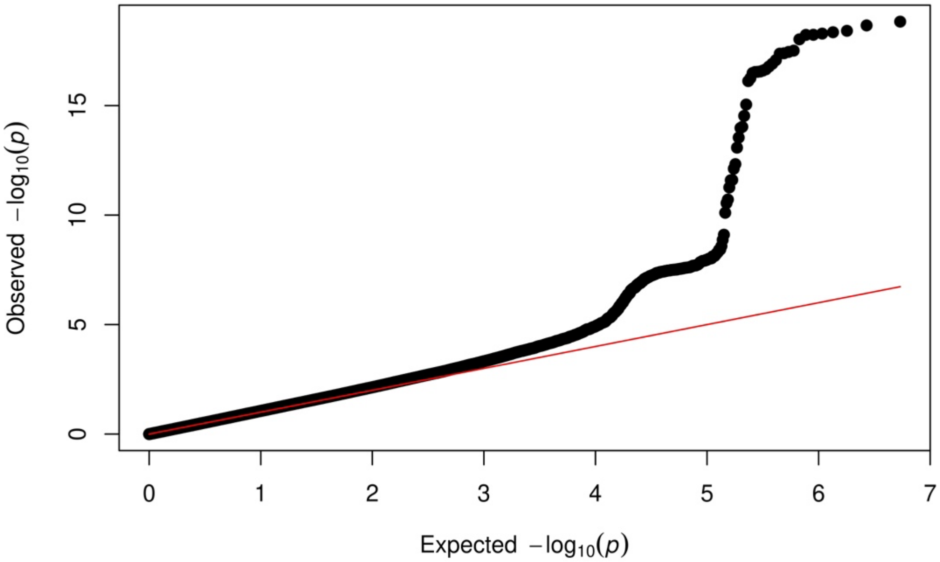

Figure S2. QQ Plot of GWAS results. This figure shows the QQ plot for the genome-wide association results from Equation (2).

Figure S3

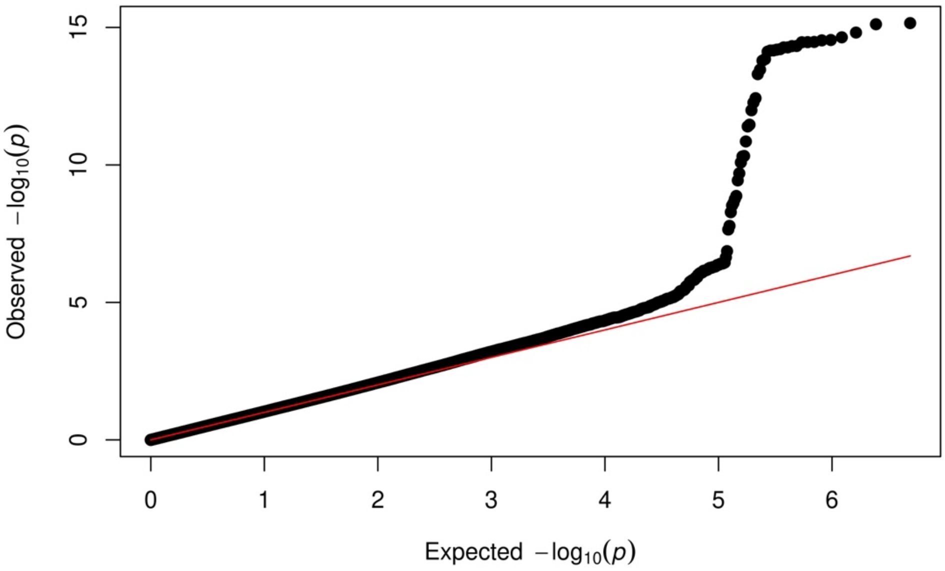

Figure S3. QQ Plot of G+E GWAS results. This figure shows the QQ plot for the genome-wide association results with ACE included as an environmental covariate. This equation is listed as Equation (3).

Figure S4

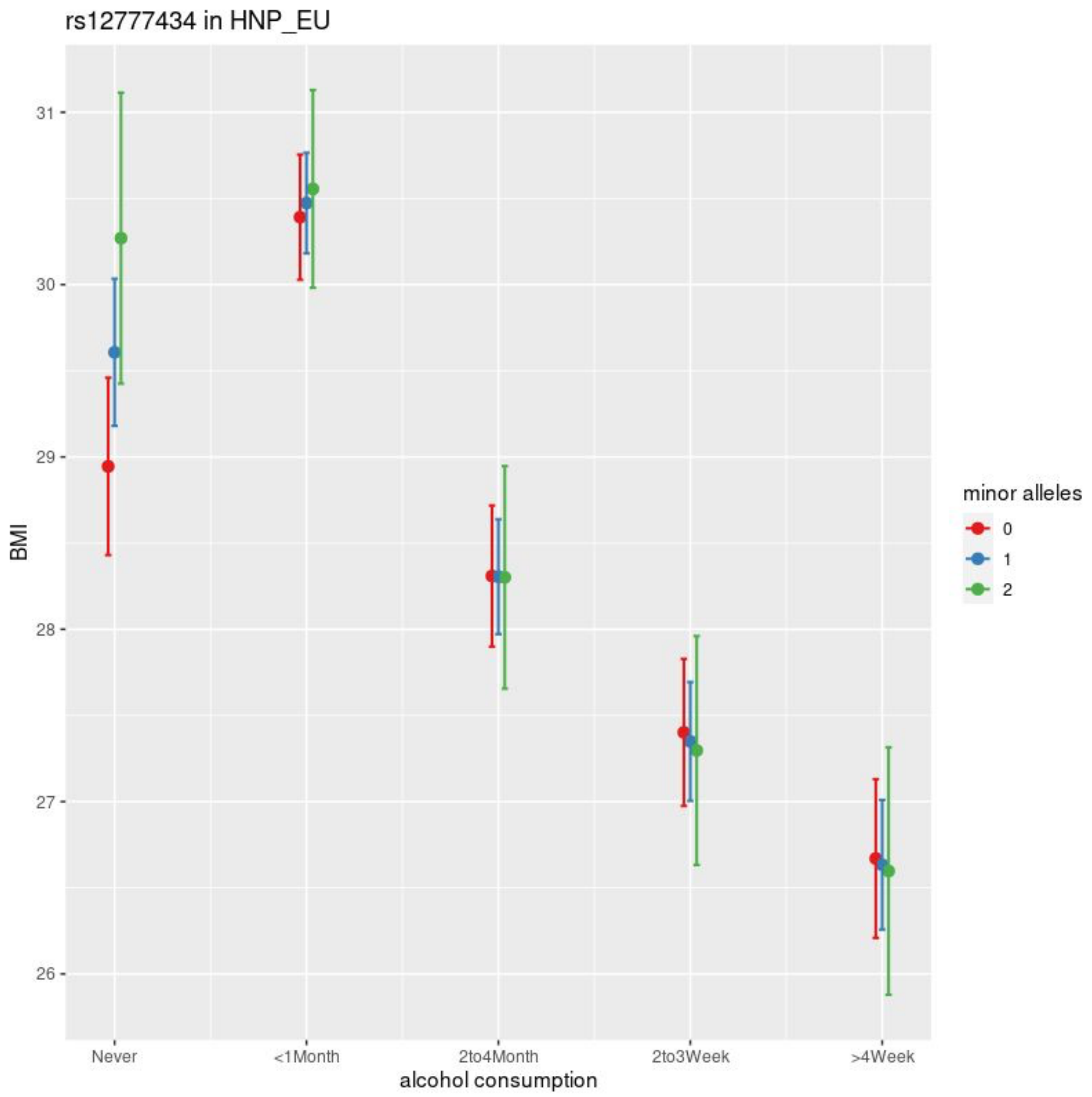

Figure S4. Interaction between rs12777434 and alcohol consumption. The minor allele of variant rs122777434 presents statistically significant associations with notably higher BMI levels in HNP_{EU} Never Drinkers (p=0.03), whereas the variant has no effect on BMI in any other group of alcohol consumption.

Figure S5

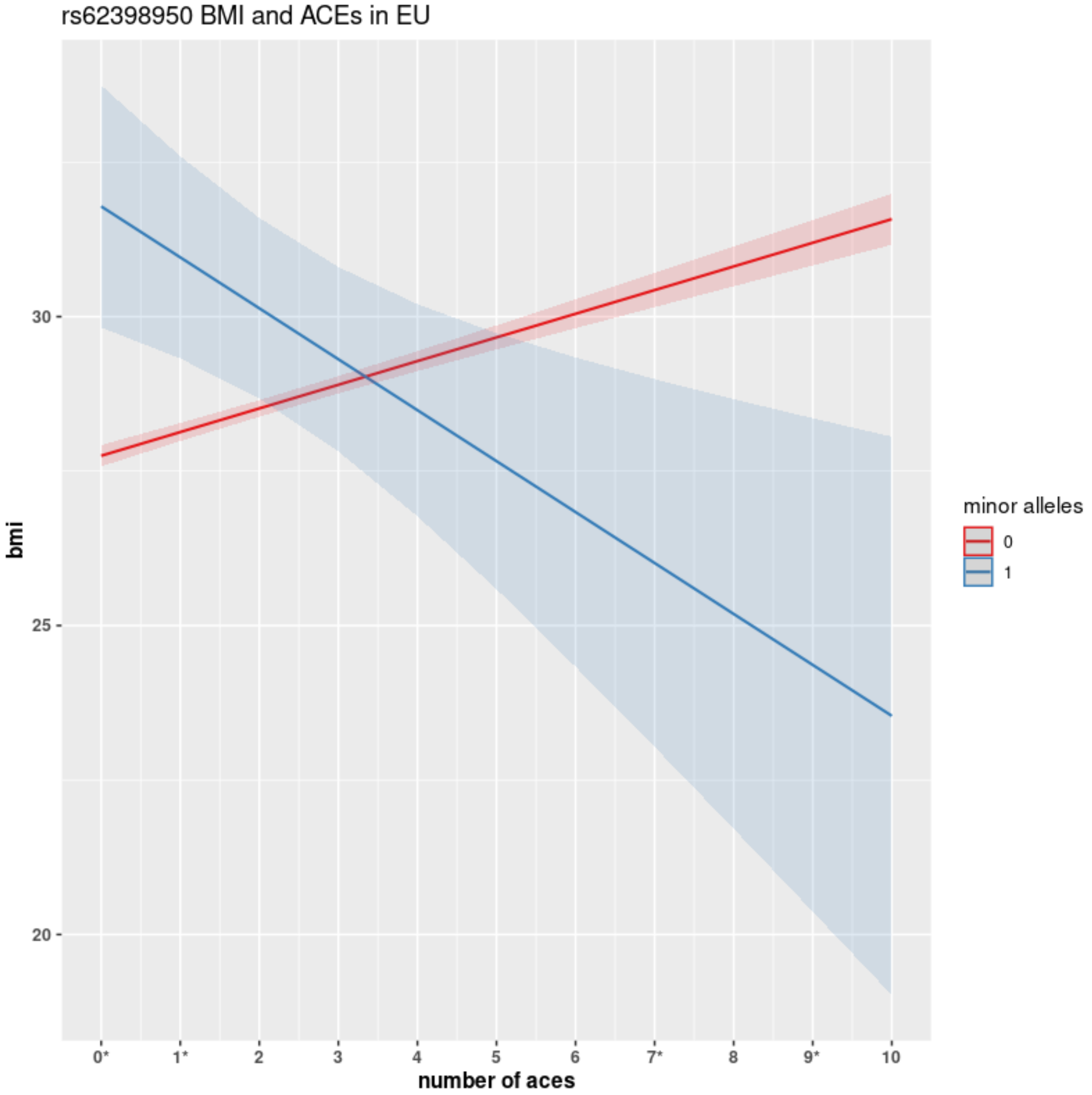

Figure S5. Interaction between rs62398950 and ACEs. Homozygotes in the reference allele show an increase in BMI for each number of ACEs encountered. However, heterozygotes show a consistent decrease in BMI values, indicating a protective effect of the allele. Statistical differences at the α=0.05 level of BMI values across genotypes occur at $N = 0$, 1, 7, and 9 ACEs, and are denoted with asterisks on the x-axis.

Figure S6

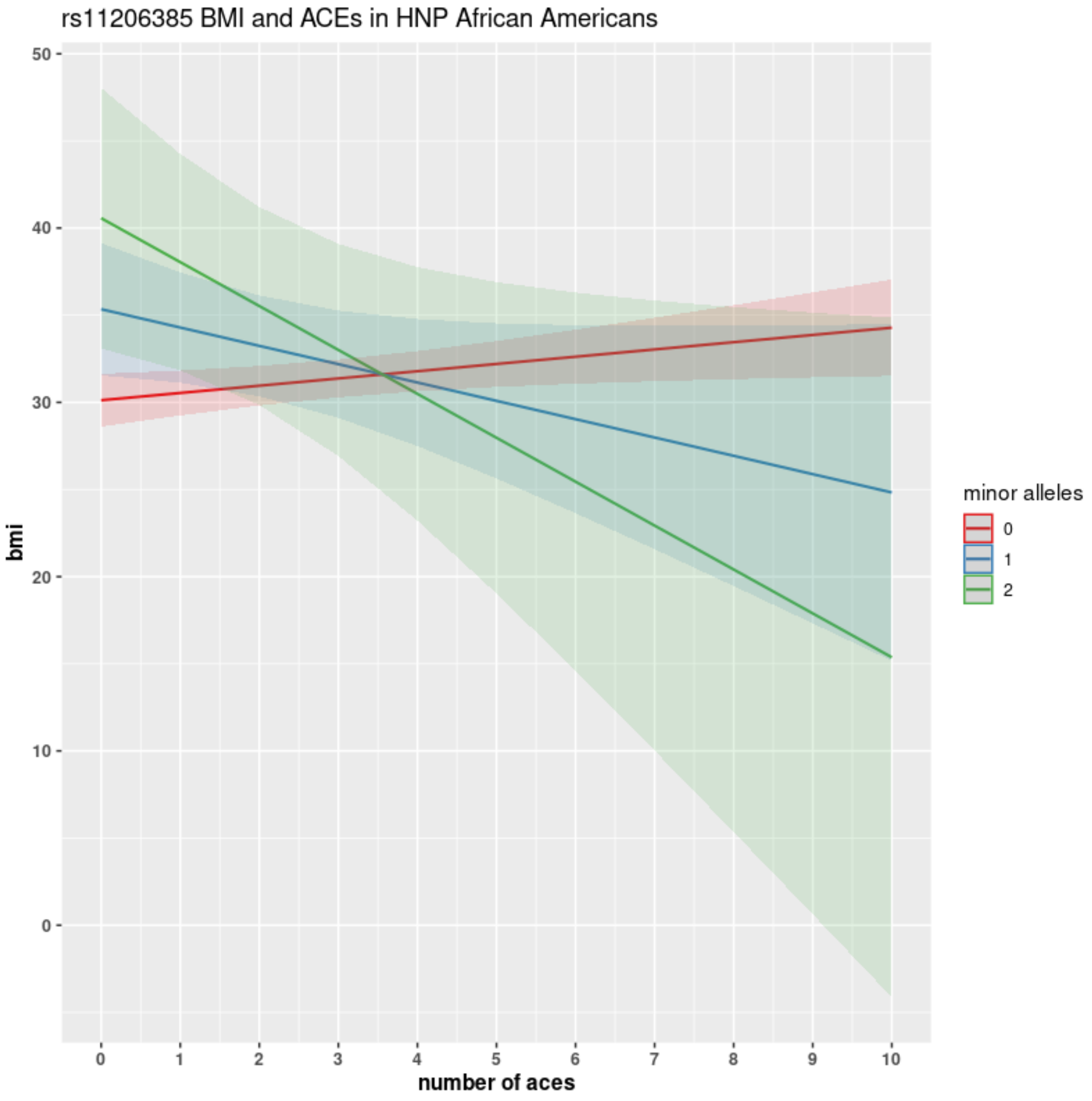

Figure S6. Interaction between rs11206385 and ACEs in the HNP_{AA}. Homozygotes in the reference allele show an increase in BMI for each number of ACEs encountered. However, with each copy of the minor allele, participants show a substantial decrease in BMI indicating a protective effect for the allele.

Figure S7

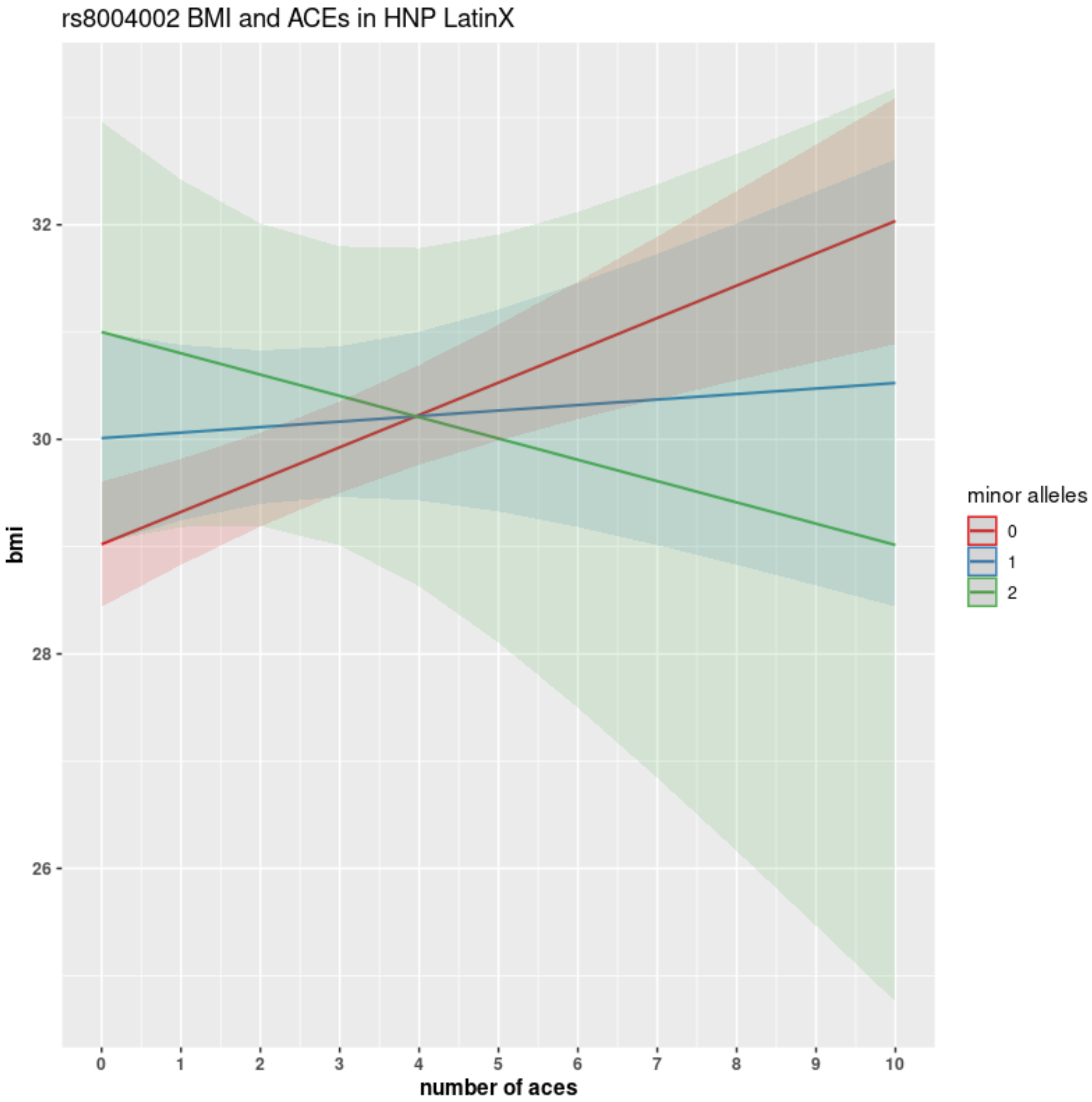

Figure S7. Interaction between rs8004002 and ACEs in the HNPLX. Homozygotes in the reference allele show a substantial increase in BMI for each number of ACEs encountered. Participants with one copy of the alternative allele do not seem to be influenced notably across the number of ACEs. However, homozygotes in the alternative allele show a show a substantial decrease in BMI, indicating a protective effect for the allele.

Figure S8

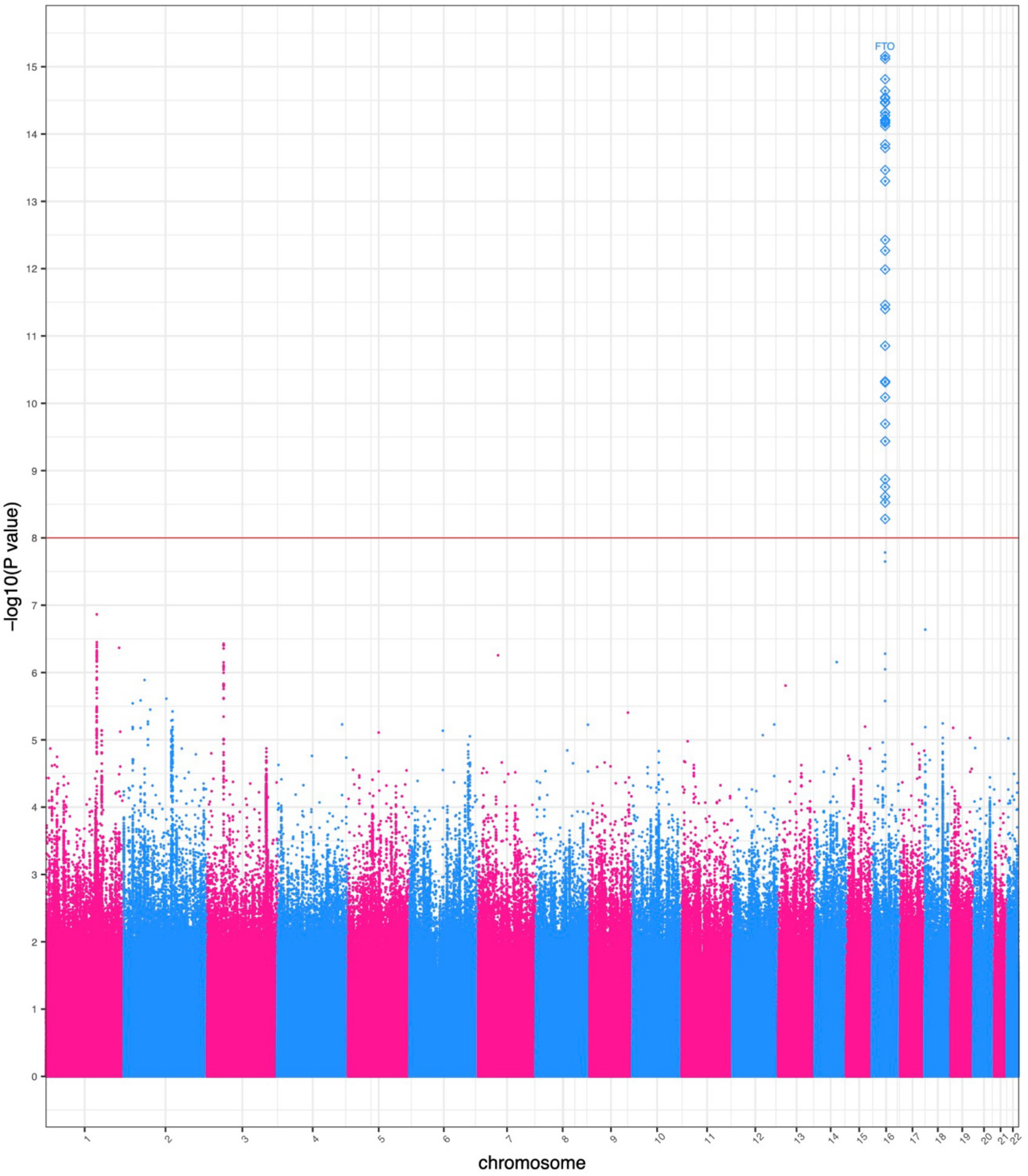

Figure S8. GWAS association results with ACEs as a covariate. Each point in this figure represents a result of a single variant in the genome-wide association study. The *x*-axis represents the genomic position of 4,876,698 variants. The *y*-axis represents -$\log_{10}$-transformed raw *p*-values of each genotypic association. For ease of viewing, only variants within genes above the horizontal line $\alpha = 1x10^{-8}$ are annotated. Note that all significant variants are in the *FTO* gene.

Figure S9

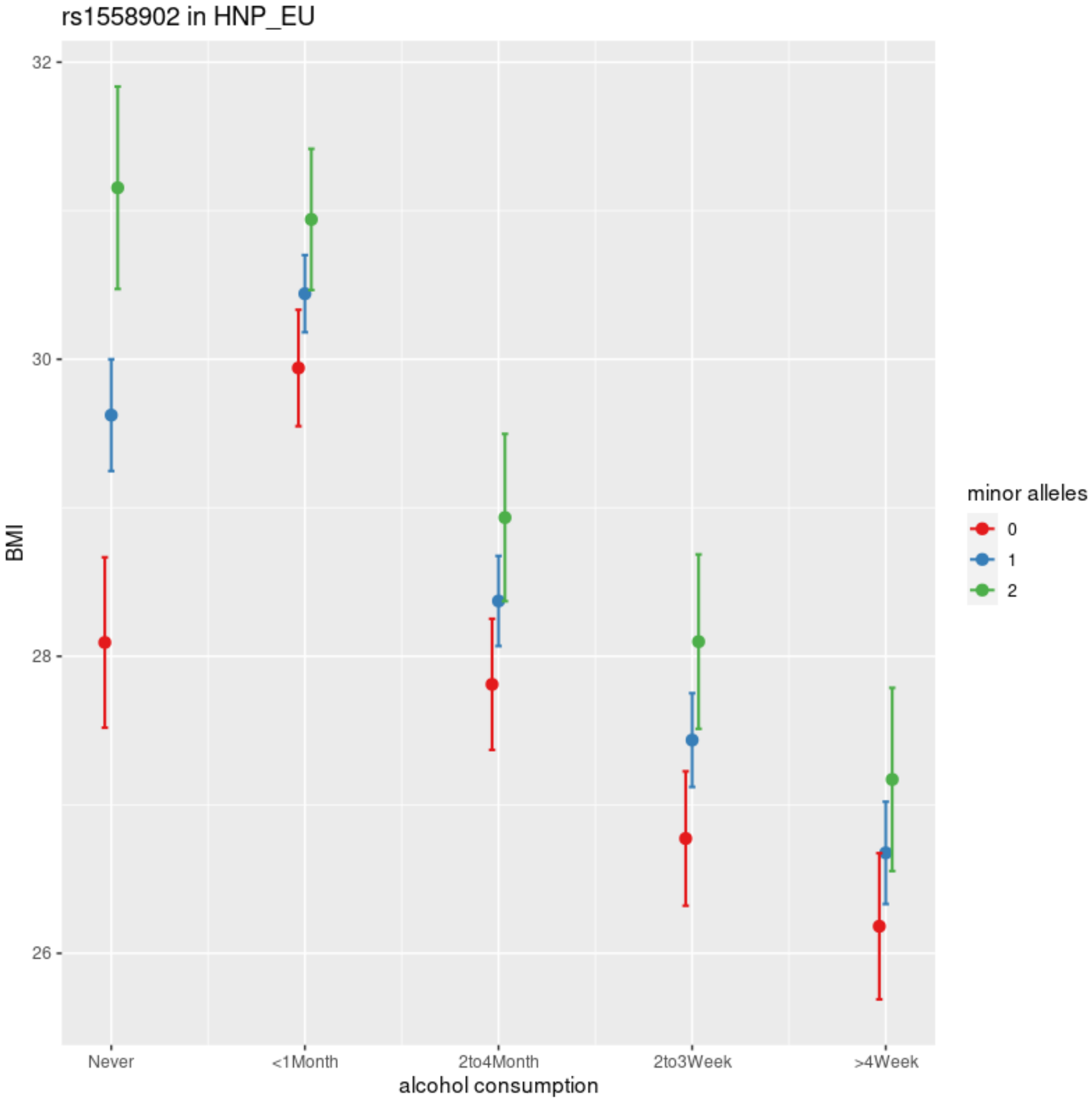

Figure S9. Interaction between rs1558902 and alcohol consumption. This figure shows how alcohol consumption patterns modify the effect of the *FTO* variant rs1558902 on BMI: minor allele carriers who drank more frequently had a much lower BMI, whereas the Never Drinkers had a greater BMI. This HNP_{EU} result with p=0.012 follows that of Rask-Anderson's study.

Figure S10

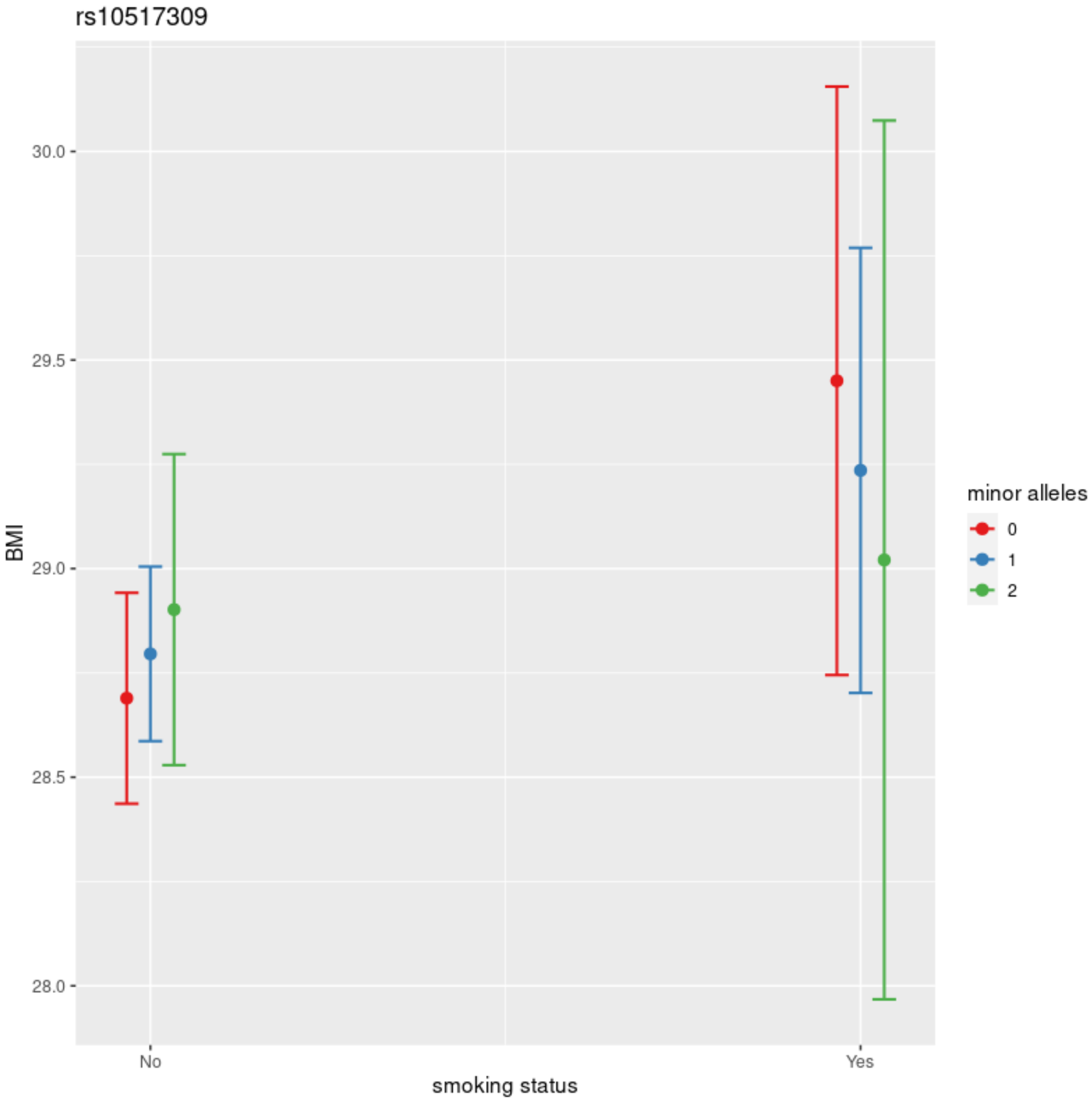

Figure S10. Interaction between rs10517309 and smoking. This figure shows how the smoking patterns modify the effect of the variant rs10517309. The minor allele of rs10517309 was associated with increased BMI in non-smokers and decreased BMI in smokers.

Table S1

Table S1. All raw and transformed BMI values for all participants are presented in this table.

Table S2

Table S2. European GWEIS results of genotype and ACE interaction

Variant	Chrom	BP	rsID	Gene	VEP Consequence	Ref	Alt	Tested	N	Beta GxE	SE	p-value GxE	MAF	Beta SNP	N(G)	Beta ACEs	N(GxE)
chr1:54553932:G:A	chr1	54553932	rs115847516	ACOT11	intron_variant	G	A	A	10894	-0.074	0.016	5.41E-06	0.034	0.18	3716	0.06	4267
chr1:54556186:G:A	chr1	54556186	rs11206385	ACOT11	intron_variant	G	A	A	10916	-0.072	0.015	3.17E-06	0.037	0.17	3807	0.06	4121
chr1:54566373:C:G	chr1	54566373	rs544101	ACOT11	intron_variant	C	G	G	11272	-0.062	0.014	5.37E-06	0.048	0.1	8584	0.06	4340
chr1:109384074:TG:T	chr1	109384075	rs142229623	SORT1	intron_variant	TG	T	T	11403	-0.1	0.022	6.25E-06	0.018	0.21	5031	0.06	4304
chr1:233942404:A:C	chr1	233942404	rs114398837	SLC35F3	intron_variant	A	C	C	11549	0.229	0.05	4.14E-06	0.003	-0.48	5691	0.05	4865
chr1:233942523:A:G	chr1	233942523	rs115036819	SLC35F3	intron_variant	A	G	G	11635	0.232	0.05	3.55E-06	0.003	-0.49	5120	0.05	4444
chr2:7519809:A:G	chr2	7519809	rs10174322	NA	NA	G	A	G	11306	0.026	0.006	8.53E-06	0.474	-0.13	927	0.05	4427
chr2:111725374:T:C	chr2	111725374	rs3853088	NA	NA	T	C	C	11192	-0.066	0.015	6.10E-06	0.040	-0.08	15965	0.05	4581
chr2:111730927:C:A	chr2	111730927	rs3893271	NA	NA	C	A	A	11414	-0.065	0.015	8.54E-06	0.039	0.17	3619	0.06	4806
chr2:111734120:G:A	chr2	111734120	rs11122911	NA	NA	G	A	A	11446	-0.066	0.014	4.54E-06	0.040	-0.06	28384	0.04	4601
chr2:122735816:C:T	chr2	122735816	rs76806574	NA	NA	C	T	T	8033	-0.215	0.044	9.98E-07	0.007	-0.06	163798	0.04	2498
chr2:234084331:C:T	chr2	234084331	rs250957	NA	non_coding_transcript_exon_variant	C	T	T	5984	-0.135	0.029	2.36E-06	0.024	-0.05	66741	0.04	1794
chr4:22586708:G:A	chr4	22586708	rs73247169	NA	NA	G	A	A	11365	0.07	0.015	2.54E-06	0.039	-0.06	29082	0.04	4172
chr4:25868546:A:T	chr4	25868546	rs12510782	LOC102723733	intron_variant	A	T	T	11472	0.057	0.013	4.79E-06	0.056	-0.06	20722	0.04	4481
chr4:183454518:A:C	chr4	183454518	rs6857406	NA	NA	A	C	C	9747	-0.09	0.019	3.38E-06	0.026	0.41	928	0.05	3746
chr5:52411576:A:C	chr5	52411576	rs113826192	NA	NA	A	C	C	11232	0.148	0.033	8.49E-06	0.008	0.16	19313	0.06	4366
chr5:113773227:G:A	chr5	113773227	rs186352945	LOC105379127	intron_variant	G	A	A	10501	-0.121	0.027	6.39E-06	0.012	-0.1	33935	0.05	4552
chr5:162384481:T:G	chr5	162384481	rs62398950	NA	NA	T	G	G	11840	-0.209	0.047	8.05E-06	0.004	0.16	35801	0.06	4069
chr6:116632203:A:G	chr6	116632203	rs751056318	RSPH4A	splice_region_variant	A	G	G	11873	-0.2	0.045	8.08E-06	0.003	0.04	820048	0.06	6362
chr6:150177056:C:T	chr6	150177056	rs75226630	PPP1R14C	intron_variant	C	T	T	8766	-0.961	0.216	8.87E-06	0.001	0.06	>1m	0.06	1371
chr7:16862061:C:T	chr7	16862061	rs140779232	AGR3	splice_acceptor_variant	C	T	T	11830	0.299	0.061	1.19E-06	0.002	-0.12	151675	0.05	4758
chr7:63211768:G:A	chr7	63211768	rs9770820	NA	non_coding_transcript_exon_variant	A	G	G	6517	-0.197	0.043	4.70E-06	0.007	-0.11	44152	0.05	2684
chr7:149252726:G:A	chr7	149252726	rs141430858	ZNF212	missense_variant	G	A	A	11870	-0.289	0.064	7.16E-06	0.002	-0.23	30981	0.05	3825
chr8:40018458:G:A	chr8	40018458	rs138192365	NA	downstream_gene_variant	G	A	A	11849	-0.054	0.012	5.70E-06	0.057	-0.23	1374	0.05	4858
chr8:40024172:G:A	chr8	40024172	rs16888636	NA	NA	G	A	A	8446	-0.066	0.014	4.19E-06	0.054	0.22	1581	0.06	3407
chr8:98171981:G:A	chr8	98171981	rs73281852	NA	NA	G	A	A	8822	-0.093	0.019	1.61E-06	0.027	-0.3	1644	0.04	3346
chr9:5523308:C:G	chr9	5523308	rs75889070	PDCD1LG2	intron_variant	C	G	G	10823	-0.162	0.034	2.21E-06	0.009	0.14	24322	0.06	3524
chr9:5523850:C:T	chr9	5523850	rs75522617	PDCD1LG2	intron_variant	C	T	T	10793	-0.161	0.036	7.01E-06	0.008	-0.86	641	0.05	3544
chr9:136014994:C:CT	chr9	136014994	rs200353628	NACC2_LOC105376322	intron_variant	CT	C	C	10498	0.09	0.02	9.14E-06	0.020	1.21	136	0.05	4649
chr10:12790651:T:TA	chr10	12790651	rs1323423426	CAMK1D	intron_variant	T	TA	TA	11241	0.027	0.006	2.58E-06	0.414	-0.04	10107	0.03	4315
chr10:12793447:T:C	chr10	12793447	rs12777434	CAMK1D	intron_variant	T	C	C	11157	0.032	0.006	1.24E-07	0.330	0.15	785	0.06	3272
chr10:12793448:G:T	chr10	12793448	rs71477259	CAMK1D	intron_variant	G	T	T	11146	0.032	0.006	1.73E-07	0.330	0.16	689	0.06	3267
chr10:17310815:T:A	chr10	17310815	rs12248753	NA	downstream_gene_variant	T	A	A	11574	0.074	0.016	6.17E-06	0.031	0.16	5084	0.06	4588
chr10:67106188:G:C	chr10	67106188	rs78687515	CTNNA3	intron_variant	G	C	C	11156	0.056	0.012	7.88E-06	0.057	0.41	430	0.05	4438
chr10:95877913:T:C	chr10	95877913	rs111750749	ENTPD1-AS1	intron_variant	T	C	C	11828	-0.079	0.018	9.31E-06	0.026	0.28	1958	0.05	4793
chr10:124582559:T:TA	chr10	124582560	rs71026101	LHPP	intron_variant	T	TA	TA	11780	0.033	0.007	9.39E-06	0.186	-0.3	284	0.05	4477
chr10:124583679:C:T	chr10	124583679	rs36096707	LHPP	intron_variant	C	T	T	11689	0.033	0.007	9.82E-06	0.184	-0.07	5337	0.05	4643
chr10:124604852:G:T	chr10	124604852	rs11245311	LHPP	intron_variant	G	T	T	11048	0.036	0.008	5.70E-06	0.165	-0.1	2845	0.05	4241
chr10:124605656:C:G	chr10	124605656	rs12771611	LHPP	intron_variant	C	G	G	8200	0.04	0.009	7.78E-06	0.174	0.13	1615	0.05	3289
chr11:9328113:CT:C	chr11	9328113	rs1391302023	NA	NA	C	CT	CT	11443	0.047	0.01	2.67E-06	0.091	0.31	489	0.05	4093
chr11:84677191:T:C	chr11	84677191	rs17147497	DLG2	intron_variant	T	C	C	11786	-0.307	0.068	7.20E-06	0.002	0.4	16157	0.05	5347
chr12:54128048:G:A	chr12	54128048	rs79424204	SMUG1	intron_variant	G	A	A	11382	-0.091	0.02	4.50E-06	0.023	0.62	450	0.05	4046
chr12:73967123:TTC:T	chr12	73967128	rs149262650	NA	NA	TTC	T	T	11496	0.087	0.018	1.25E-06	0.024	0.95	180	0.06	4058
chr13:38970772:C:G	chr13	38970772	rs77744003	STOML3	intron_variant	C	G	G	11359	-0.077	0.017	5.60E-06	0.029	-0.61	367	0.05	4448
chr14:22633879:A:G	chr14	22633879	rs1753429	OR6J1	synonymous_variant	A	G	G	11054	-0.033	0.007	4.92E-06	0.209	0.38	161	0.05	4074
chr14:22633977:C:A	chr14	22633977	rs1681596	OR6J1	missense_variant	C	A	A	9074	-0.039	0.008	1.45E-06	0.213	0.48	98	0.05	2789
chr14:32715684:G:A	chr14	32715684	rs8004002	AKAP6	intron_variant	G	A	A	11360	0.053	0.011	1.90E-06	0.071	0.11	4906	0.06	4071
chr14:32724407:C:T	chr14	32724407	rs7141440	AKAP6	intron_variant	C	T	T	11248	0.054	0.012	6.41E-06	0.064	0.11	5410	0.06	4327
chr15:42791296:G:C	chr15	42791296	rs79737031	TTBK2	intron_variant	G	C	C	11868	0.17	0.038	6.31E-06	0.006	0.22	12747	0.06	4129
chr15:42791672:G:A	chr15	42791672	rs12595722	TTBK2	intron_variant	G	A	A	11870	0.161	0.036	9.09E-06	0.007	0.23	11484	0.05	4560
chr15:101874151:G:A	chr15	101874151	rs145351074	NA	upstream_gene_variant	G	A	A	10477	-0.123	0.025	5.55E-07	0.014	0.24	4830	0.05	3586
chr17:36832861:A:C	chr17	36832861	rs58352591	NA	NA	A	C	C	4314	0.11	0.022	5.64E-07	0.047	-0.19	2404	0.05	1384
chr18:39749772:A:G	chr18	39749772	rs78700182	MIR924HG	intron_variant	A	G	G	11747	-0.078	0.018	7.65E-06	0.026	-0.06	42883	0.03	4991
chr19:29588839:T:A	chr19	29588839	rs80211484	NA	NA	T	A	A	7090	0.261	0.058	6.29E-06	0.003	-0.06	376994	0.03	3912
chr19:46303928:C:T	chr19	46303928	rs142517237	HIF3A	synonymous_variant	C	T	T	11878	-0.653	0.144	6.29E-06	0.001	-0.06	>1m	0.03	3618

**Note that the BETA and SE values are based on the Rank Inverse Normalized BMI values

Table S2. GWEIS results with the ACE environmental interaction.

Table S3

Table S3. G-Only significant hits from the GWAS without ACE as a covariate.

Table S4

Table S4. European GWAS results inculding ACEs as environmental covariate

Variant	Chrom	BP	rsID	Gene	Ref	Alt	Tested	MAF	N	Beta(G)	SE(G)	Pval(G)	Beta(E)	SE(E)	Pval(E)
chr16:53765366:G:GT	chr16	53765366	rs572635656/rs199952722	FTO	G	GT	GT	0.4499	10960	0.105	0.014	1.425E-14	0.052	0.004	5.71E-35
chr16:53765367:G:T	chr16	53765367	rs369160745/rs57292959/rs143429070	FTO	G	T	T	0.4501	10886	0.109	0.014	1.5364E-15	0.053	0.004	1.98E-35
chr16:53765595:G:A	chr16	53765595	rs9937053	FTO	G	A	A	0.4028	7913	0.098	0.016	1.3412E-09	0.046	0.005	1.27E-20
chr16:53765935:G:A	chr16	53765935	rs9937354	FTO	G	A	A	0.4224	9474	0.103	0.014	1.0279E-12	0.051	0.005	1.28E-29
chr16:53765993:A:G	chr16	53765993	rs9928094	FTO	A	G	G	0.4353	11796	0.101	0.013	7.5701E-15	0.052	0.004	7.30E-38
chr16:53766065:T:G	chr16	53766065	rs9930333	FTO	T	G	G	0.4346	11844	0.101	0.013	6.8453E-15	0.052	0.004	1.18E-37
chr16:53766073:T:A	chr16	53766073	rs9930397	FTO	T	A	A	0.4346	11855	0.101	0.013	6.9015E-15	0.052	0.004	1.18E-37
chr16:53766656:G:A	chr16	53766656	rs9939973	FTO	G	A	A	0.4352	11863	0.101	0.013	5.3278E-15	0.052	0.004	6.23E-38
chr16:53766717:C:G	chr16	53766717	rs9940646	FTO	C	G	G	0.4351	11877	0.101	0.013	5.321E-15	0.052	0.004	1.31E-37
chr16:53766842:G:A	chr16	53766842	rs9940128	FTO	G	A	A	0.4351	11880	0.101	0.013	4.7849E-15	0.052	0.004	1.30E-37
chr16:53767637:C:T	chr16	53767637	rs9923147	FTO	C	T	T	0.4343	11636	0.103	0.013	2.8602E-15	0.053	0.004	5.20E-38
chr16:53769275:A:T	chr16	53769275	rs1558901	FTO	A	T	T	0.432	11442	0.100	0.013	3.4305E-14	0.052	0.004	1.24E-35
chr16:53770428:C:T	chr16	53770428	rs1861866	FTO	T	C	C	0.4842	11634	-0.081	0.013	3.6663E-10	0.053	0.004	4.95E-38
chr16:53771053:T:C	chr16	53771053	rs10852521	FTO	C	T	T	0.4841	11648	-0.078	0.013	1.749E-09	0.052	0.004	2.07E-36
chr16:53775335:G:A	chr16	53775335	rs1121980	FTO	G	A	A	0.4357	11826	0.102	0.013	4.7397E-15	0.052	0.004	2.06E-37
chr16:53776774:T:C	chr16	53776774	rs7193144	FTO	T	C	C	0.4018	10779	0.103	0.014	5.0077E-14	0.053	0.004	4.13E-35
chr16:53778702:G:A	chr16	53778702	rs8057044	FTO	G	A	A	0.485	11785	0.076	0.013	2.9827E-09	0.051	0.004	2.87E-36
chr16:53779455:T:G	chr16	53779455	rs17817449	FTO	T	G	G	0.4054	11361	0.104	0.013	6.4529E-15	0.052	0.004	2.19E-36
chr16:53779538:A:T	chr16	53779538	rs8043757	FTO	A	T	T	0.4056	11670	0.106	0.013	7.6383E-16	0.053	0.004	2.80E-38
chr16:53782363:C:A	chr16	53782363	rs8050136	FTO	C	A	A	0.4047	11704	0.106	0.013	6.9538E-16	0.052	0.004	4.98E-37
chr16:53782840:A:G	chr16	53782840	rs8051591	FTO	A	G	G	0.3691	6108	0.111	0.019	2.4315E-09	0.062	0.006	2.02E-27
chr16:53782926:G:A	chr16	53782926	rs9935401	FTO	G	A	A	0.405	11737	0.102	0.013	6.1789E-15	0.052	0.004	2.77E-37
chr16:53785257:T:C	chr16	53785257	rs9936385	FTO	T	C	C	0.4023	11612	0.102	0.013	1.6145E-14	0.051	0.004	1.11E-35
chr16:53785286:G:C	chr16	53785286	rs9923233	FTO	G	C	C	0.4032	11606	0.104	0.013	2.9485E-15	0.051	0.004	7.44E-36
chr16:53785965:C:T	chr16	53785965	rs11075989	FTO	C	T	T	0.3776	7797	0.103	0.016	2.0188E-10	0.051	0.005	8.33E-25
chr16:53785981:A:G	chr16	53785981	rs11075990	FTO	A	G	G	0.3835	8004	0.104	0.016	8.1341E-11	0.051	0.005	3.72E-25
chr16:53786025:A:T	chr16	53786025	rs11075991	FTO	A	T	T	0.3839	8030	0.107	0.016	1.4001E-11	0.051	0.005	5.57E-25
chr16:53786591:G:A	chr16	53786591	rs9926289	FTO	G	A	A	0.4045	11873	0.103	0.013	3.4488E-15	0.052	0.004	1.57E-37
chr16:53786615:T:A	chr16	53786615	rs9939609	FTO	T	A	A	0.4046	11875	0.103	0.013	2.2837E-15	0.052	0.004	1.44E-37
chr16:53787703:A:G	chr16	53787703	rs7202116	FTO	A	G	G	0.3941	11146	0.099	0.014	3.7416E-13	0.051	0.004	2.58E-34
chr16:53788257:AT:A	chr16	53788257	rs113935429	FTO	AT	A	A	0.3514	10555	0.093	0.014	4.9007E-11	0.052	0.004	9.05E-34
chr16:53788325:A:G	chr16	53788325	rs1243617223/rs62033403/rs386790837	FTO	A	G	G	0.4016	11687	0.103	0.013	3.3576E-15	0.052	0.004	2.27E-37
chr16:53788327:A:G	chr16	53788327	rs62033404/rs1567986734	FTO	A	G	G	0.4016	11685	0.103	0.013	3.4169E-15	0.052	0.004	2.54E-37
chr16:53788739:A:G	chr16	53788739	rs1417860482/rs7185735	FTO	A	G	G	0.3883	9757	0.105	0.015	5.406E-13	0.053	0.004	2.11E-32
chr16:53791576:C:T	chr16	53791576	rs9941349	FTO	C	T	T	0.415	10867	0.094	0.014	3.9933E-12	0.052	0.004	3.26E-34
chr16:53796540:A:G	chr16	53796540	rs9930501	FTO	A	G	G	0.3884	9327	0.089	0.015	5.2324E-09	0.050	0.005	7.51E-28
chr16:53796553:A:G	chr16	53796553	rs9930506	FTO	A	G	G	0.4083	10344	0.094	0.014	4.7295E-11	0.051	0.004	8.12E-32
chr16:53796579:T:C	chr16	53796579	rs9932754	FTO	T	C	C	0.4085	9943	0.101	0.015	3.445E-12	0.051	0.004	4.12E-30

**Note that the Beta(G) and SE(G) are based on the Rank Inverse Normalized BMI values

***Results are based on a significance threshold of FDR = 6.09x10-7

Table S4. G+E GWAS results including ACEs as an environmental covariate. Note that all hits are in the *FTO* gene

Table S5

Table S5. Distribution of ethnicities in the HNP

Ethnicity	N	(%)	Mean BMI	Mean Number of ACEs
African American	304	1.92%	31.08	2.69
East Asian	367	2.31%	25.7	1.48
European	12,939	80.92%	28.69	2.01
LatinX	1774	11.18%	29.76	2.59
South Asian	528	3.33%	25.96	1.44
Other	54	0.34%	28.62	2.31

**This cohort represents the 15,866 participants who had BMI records and recalled ACE experiences.

Table S5. Distribution of ethnicities across the HNP.

Table S6

Table S6. BMI and ACE covariate regression.

	Estimate	Std. Error	t value	Pr(>|t|)
(Intercept)	29.71	0.41	72.91	0.000000000000
ACEs	0.35	0.02	15.56	0.000000000000
Age	0.01	0.00	1.99	0.047156222140
Sex (Male)	0.42	0.11	3.67	0.000244247399
Ethnicity (EastAsian)	-4.99	0.50	-9.96	0.000000000000
Ethnicity (European)	-2.21	0.38	-5.89	0.000000003979
Ethnicity (LatinX)	-1.26	0.40	-3.15	0.001648253107
Ethnicity (Other)	-2.32	0.46	-4.99	0.000000594732
Ethnicity (SouthAsian)	-4.68	0.95	-4.91	0.000000917737

Table S6. Results of BMI and ACE covariate regression.
